# Supplementary material for: Simulating multiple variability in spatially resolved transcriptomics with scCube
Source: Nat Commun. 2024 Jun 12;15:5021. doi: 10.1038/s41467-024-49445-0 (PMC11169532; doi:10.1038/s41467-024-49445-0)
Supplement: Supplementary file 1 — Supplementary Information [file 41467_2024_49445_MOESM1_ESM.pdf]

Simulating multiple variability in spatially resolved transcriptomics with scCube

Jingyang Qian[†], Hudong Bao[†], Xin Shao, Yin Fang, Jie Liao, Zhuo Chen, Chengyu Li, Wenbo Guo, Yining Hu, Anyao Li, Yue Yao, Xiaohui Fan[*], & Yiyu Cheng[*]

Supplementary Figures

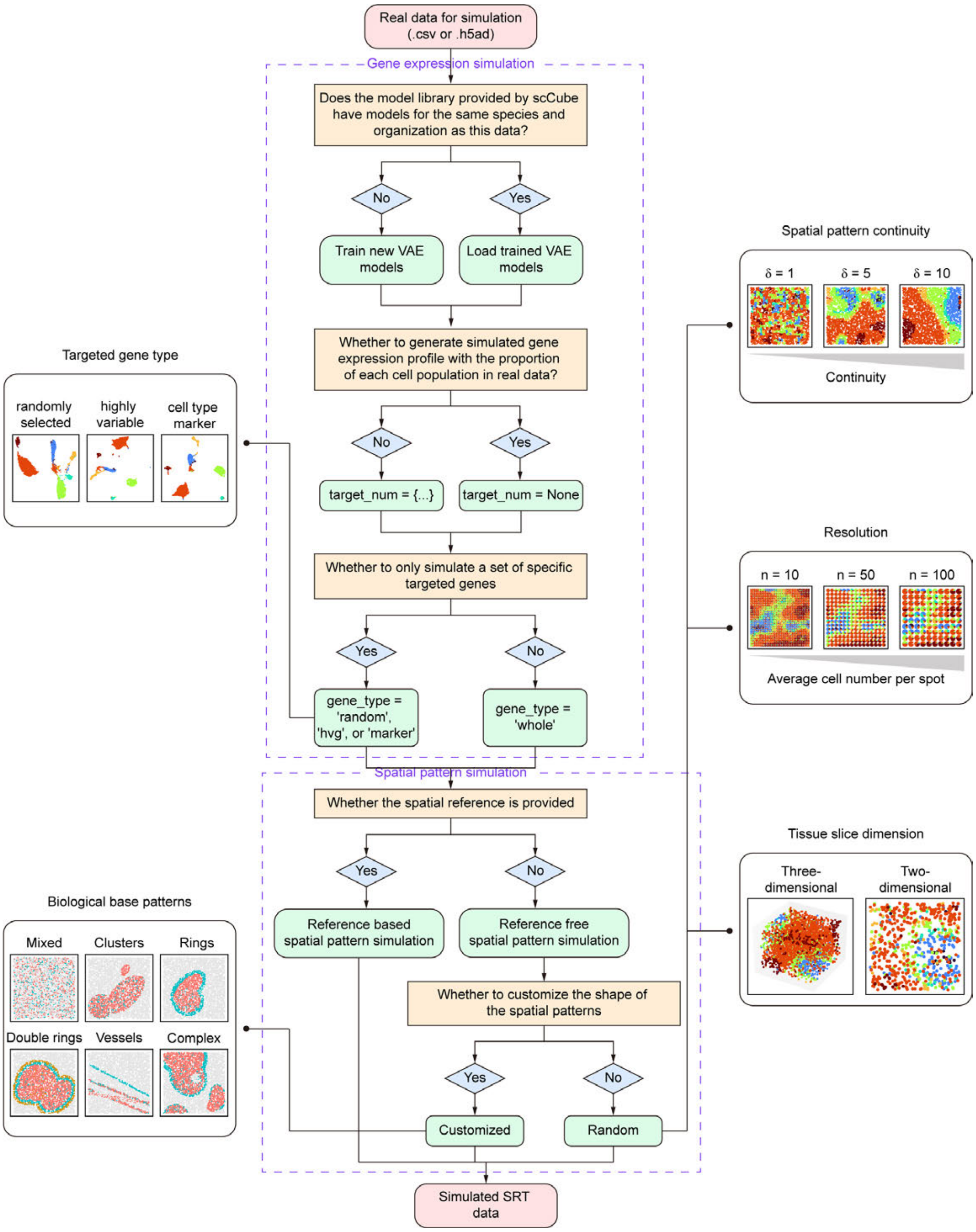

Supplementary Figure S1. Flow diagram to simulate various SRT data by scCube.

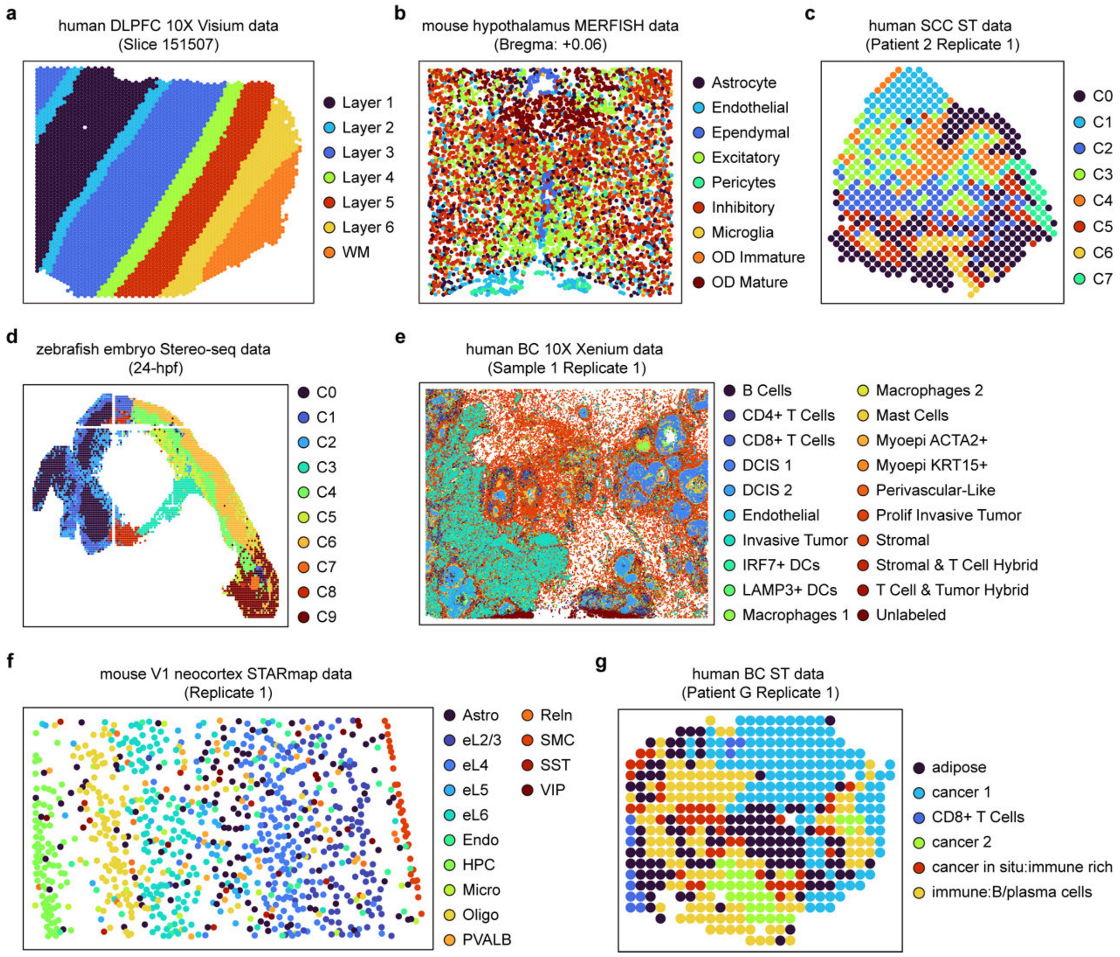

Supplementary Figure S2. The real SRT datasets utilized in simulation performance comparison of scCube with other simulators. a, The human DLPFC 10X Visium data (Slice 151507). **b,** The mouse hypothalamus MERFISH data (Bregma: +0.06). **c,** The human SCC ST data (Patient 2 Replicate 1). **d,** The zebrafish embryo Stereo-seq data (24-hpf). **e,** The human BC 10X Xenium data (Sample 1 Replicate 1). **f,** The mouse V1 neocortex STARmap data (Replicate 1). **g,** The human BC ST data (Patient G Replicate 1).

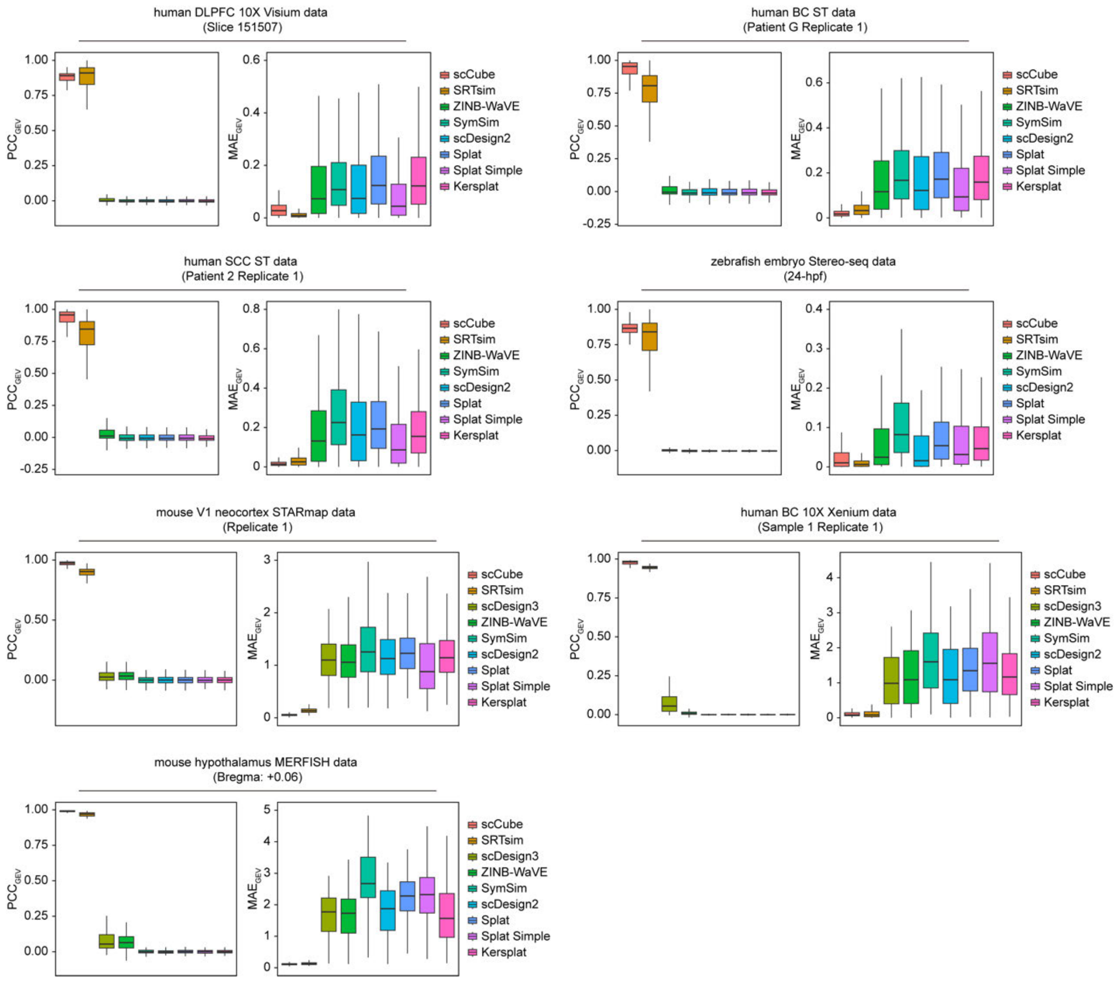

Supplementary Figure S3. Boxplots of PCC and MAE values between the gene expression vector for each gene across spatial positions in real data and simulated data generated by scCube and other simulators across seven benchmark datasets. The simulation results for all genes of scDesign3 are not provides in the Stereo-seq data and ST data due to speed constraints of the training step. Data are presented as boxplots (minima, 25th percentile, median, 75th percentile, and maxima). The number of data points are 18,094, 14,992, 16,772, 12,838, 1,020, 313, and 155 for human DLPFC 10X Visium data, human BC ST data, human SCC ST data, zebrafish Stereo-seq data, mouse V1 neocortex STARmap data, human BC 10X Xenium data, and mouse hypothalamus MERFISH data, respectively. Source data are provided as a Source Data file.

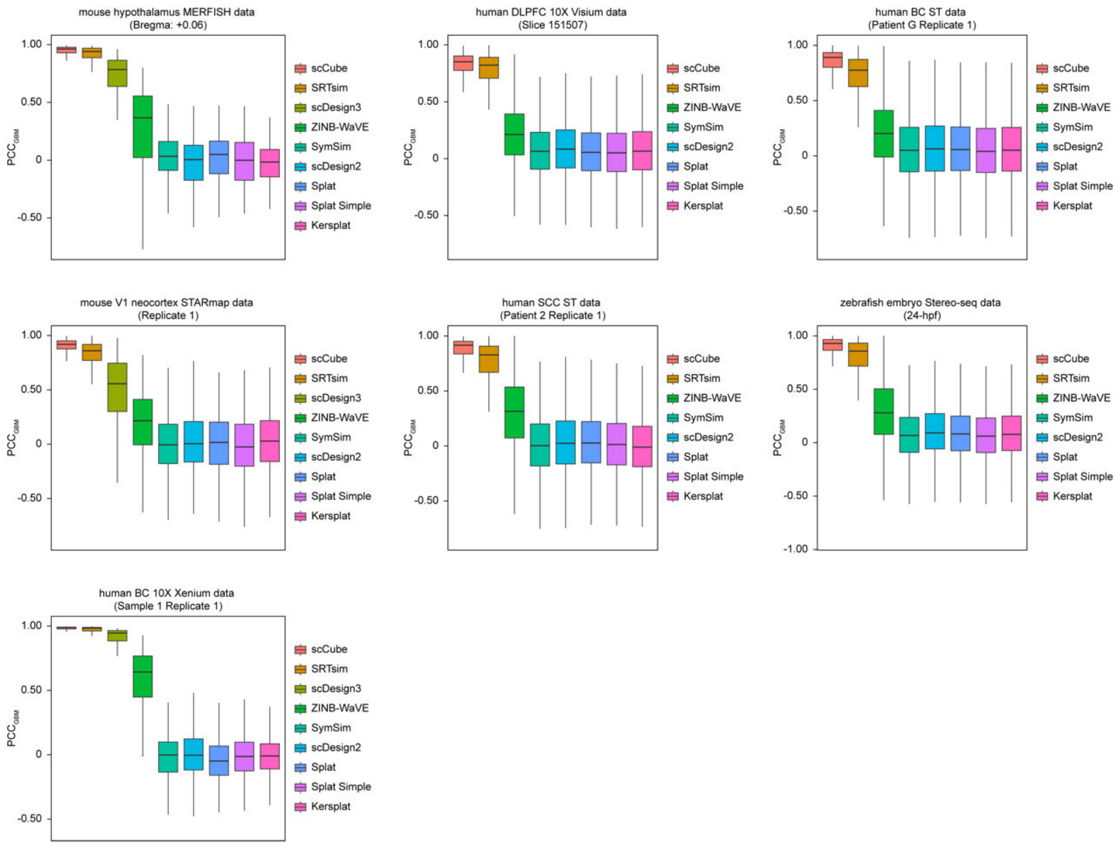

Supplementary Figure S4. Boxplots of PCC values between the gene expression value for each gene from the simulated data's spatial locations predicted by the two generalized boosted regression models (GBMs) trained on real data and simulated data generated by scCube and other simulators separately across seven benchmark datasets. The simulation results for all genes of scDesign3 are not provides in the Stereo-seq data and ST data due to speed constraints of the training step. Data are presented as boxplots (minima, 25th percentile, median, 75th percentile, and maxima). The number of data points are 155, 18,094, 14,992, 1,020, 16,772, 12,838, and 313 for mouse hypothalamus MERFISH data, human DLPFC 10X Visium data, human BC ST data, mouse V1 neocortex STARmap data, human SCC ST data, zebrafish Stereo-seq data, and human BC 10X Xenium data, respectively. Source data are provided as a Source Data file.

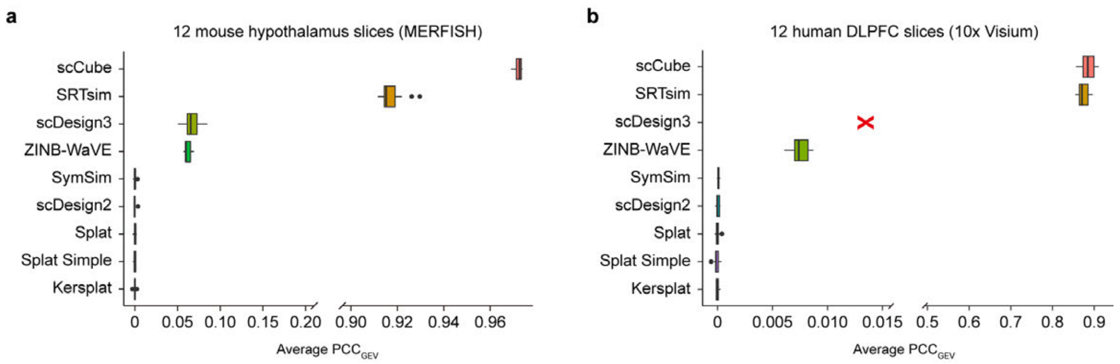

Supplementary Figure S5. Boxplots of PCC values between the gene expression vector for each gene across spatial positions in real data and simulated data generated by scCube and other simulators across all tissue slices on the mouse hypothalamus MERFISH (a) and human DLPFC 10X Visium (b) datasets. Data are presented as boxplots (minima, 25th percentile, median, 75th percentile, and maxima). The number of data points are of each method are 12 for mouse hypothalamus MERFISH data; the number of data points are of each method except scDesign3 are 12 for human DLPFC 10X Visium data. Source data are provided as a Source Data file.

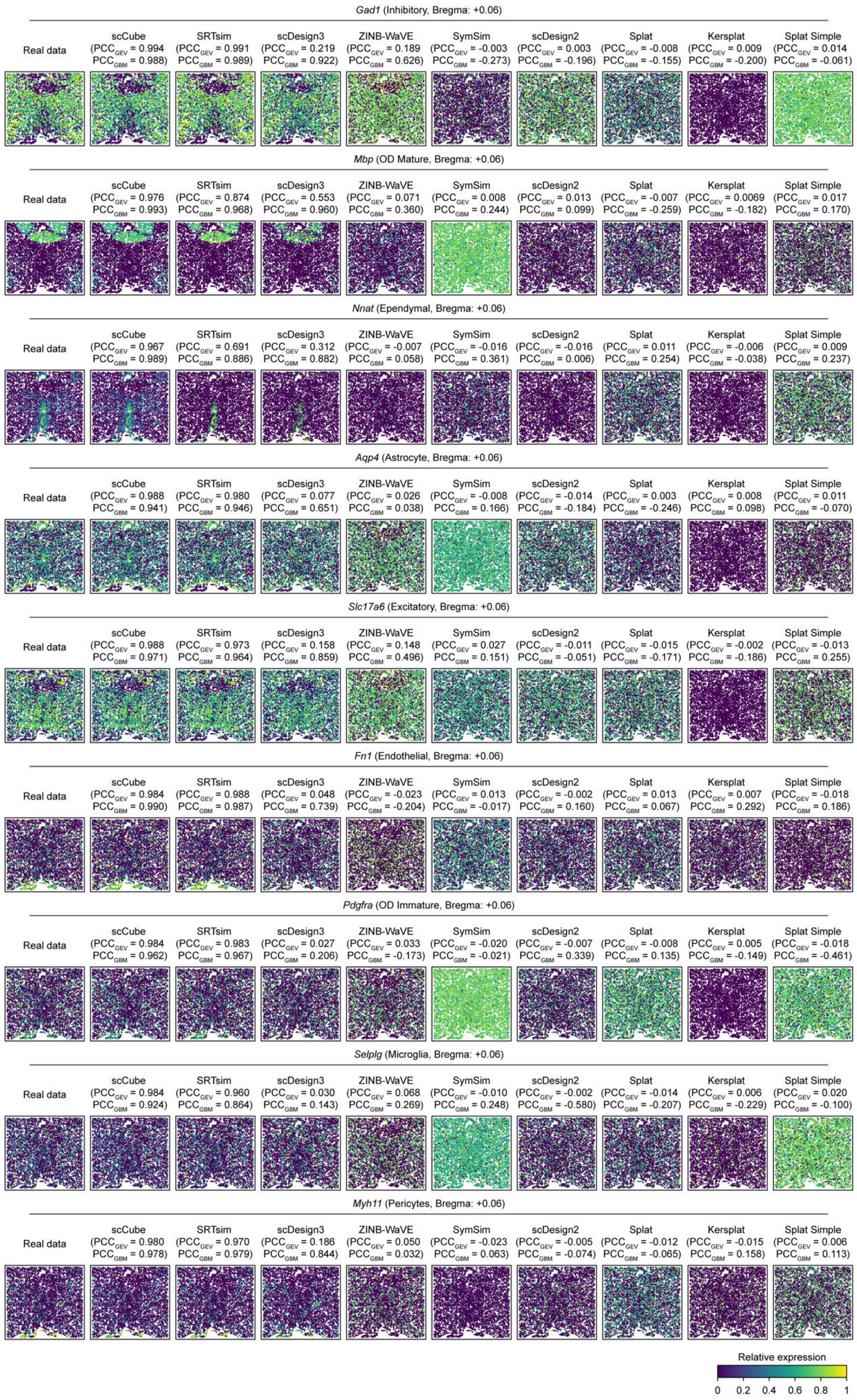

Gad1 (Inhibitory, Bregma: +0.06)
Real data
scCube (PCC$_{GEV}$ = 0.994 PCC$_{GBM}$ = 0.988)
SRTsim (PCC$_{GEV}$ = 0.991 PCC$_{GBM}$ = 0.989)
scDesign3 (PCC$_{GEV}$ = 0.219 PCC$_{GBM}$ = 0.922)
ZINB-WaVE (PCC$_{GEV}$ = 0.189 PCC$_{GBM}$ = 0.626)
SymSim (PCC$_{GEV}$ = -0.003 PCC$_{GBM}$ = -0.273)
scDesign2 (PCC$_{GEV}$ = 0.003 PCC$_{GBM}$ = -0.196)
Splat (PCC$_{GEV}$ = -0.008 PCC$_{GBM}$ = -0.155)
Kersplat (PCC$_{GEV}$ = 0.009 PCC$_{GBM}$ = -0.200)
Splat Simple (PCC$_{GEV}$ = 0.014 PCC$_{GBM}$ = -0.061)
Mbp (OD Mature, Bregma: +0.06)
Real data
scCube (PCC$_{GEV}$ = 0.976 PCC$_{GBM}$ = 0.993)
SRTsim (PCC$_{GEV}$ = 0.874 PCC$_{GBM}$ = 0.968)
scDesign3 (PCC$_{GEV}$ = 0.553 PCC$_{GBM}$ = 0.960)
ZINB-WaVE (PCC$_{GEV}$ = 0.071 PCC$_{GBM}$ = 0.360)
SymSim (PCC$_{GEV}$ = 0.008 PCC$_{GBM}$ = 0.244)
scDesign2 (PCC$_{GEV}$ = 0.013 PCC$_{GBM}$ = 0.099)
Splat (PCC$_{GEV}$ = -0.007 PCC$_{GBM}$ = -0.259)
Kersplat (PCC$_{GEV}$ = 0.0069 PCC$_{GBM}$ = -0.182)
Splat Simple (PCC$_{GEV}$ = 0.017 PCC$_{GBM}$ = 0.170)
Nnat (Ependymal, Bregma: +0.06)
Real data
scCube (PCC$_{GEV}$ = 0.967 PCC$_{GBM}$ = 0.989)
SRTsim (PCC$_{GEV}$ = 0.691 PCC$_{GBM}$ = 0.886)
scDesign3 (PCC$_{GEV}$ = 0.312 PCC$_{GBM}$ = 0.882)
ZINB-WaVE (PCC$_{GEV}$ = -0.007 PCC$_{GBM}$ = 0.058)
SymSim (PCC$_{GEV}$ = -0.016 PCC$_{GBM}$ = 0.361)
scDesign2 (PCC$_{GEV}$ = -0.016 PCC$_{GBM}$ = 0.006)
Splat (PCC$_{GEV}$ = 0.011 PCC$_{GBM}$ = 0.254)
Kersplat (PCC$_{GEV}$ = -0.006 PCC$_{GBM}$ = -0.038)
Splat Simple (PCC$_{GEV}$ = 0.009 PCC$_{GBM}$ = 0.237)
Aqp4 (Astrocyte, Bregma: +0.06)
Real data
scCube (PCC$_{GEV}$ = 0.988 PCC$_{GBM}$ = 0.941)
SRTsim (PCC$_{GEV}$ = 0.980 PCC$_{GBM}$ = 0.946)
scDesign3 (PCC$_{GEV}$ = 0.077 PCC$_{GBM}$ = 0.651)
ZINB-WaVE (PCC$_{GEV}$ = 0.026 PCC$_{GBM}$ = 0.038)
SymSim (PCC$_{GEV}$ = -0.008 PCC$_{GBM}$ = 0.166)
scDesign2 (PCC$_{GEV}$ = -0.014 PCC$_{GBM}$ = -0.184)
Splat (PCC$_{GEV}$ = 0.003 PCC$_{GBM}$ = -0.246)
Kersplat (PCC$_{GEV}$ = 0.008 PCC$_{GBM}$ = 0.098)
Splat Simple (PCC$_{GEV}$ = 0.011 PCC$_{GBM}$ = -0.070)
Slc17a6 (Excitatory, Bregma: +0.06)
Real data
scCube (PCC$_{GEV}$ = 0.988 PCC$_{GBM}$ = 0.971)
SRTsim (PCC$_{GEV}$ = 0.973 PCC$_{GBM}$ = 0.964)
scDesign3 (PCC$_{GEV}$ = 0.158 PCC$_{GBM}$ = 0.859)
ZINB-WaVE (PCC$_{GEV}$ = 0.148 PCC$_{GBM}$ = 0.496)
SymSim (PCC$_{GEV}$ = 0.027 PCC$_{GBM}$ = 0.151)
scDesign2 (PCC$_{GEV}$ = -0.011 PCC$_{GBM}$ = -0.051)
Splat (PCC$_{GEV}$ = -0.015 PCC$_{GBM}$ = -0.171)
Kersplat (PCC$_{GEV}$ = -0.002 PCC$_{GBM}$ = -0.186)
Splat Simple (PCC$_{GEV}$ = -0.013 PCC$_{GBM}$ = 0.255)
Fn1 (Endothelial, Bregma: +0.06)
Real data
scCube (PCC$_{GEV}$ = 0.984 PCC$_{GBM}$ = 0.990)
SRTsim (PCC$_{GEV}$ = 0.988 PCC$_{GBM}$ = 0.987)
scDesign3 (PCC$_{GEV}$ = 0.048 PCC$_{GBM}$ = 0.739)
ZINB-WaVE (PCC$_{GEV}$ = -0.023 PCC$_{GBM}$ = -0.204)
SymSim (PCC$_{GEV}$ = 0.013 PCC$_{GBM}$ = -0.017)
scDesign2 (PCC$_{GEV}$ = -0.002 PCC$_{GBM}$ = 0.160)
Splat (PCC$_{GEV}$ = 0.013 PCC$_{GBM}$ = 0.067)
Kersplat (PCC$_{GEV}$ = 0.007 PCC$_{GBM}$ = 0.292)
Splat Simple (PCC$_{GEV}$ = -0.018 PCC$_{GBM}$ = 0.186)
Pdgfra (OD Immature, Bregma: +0.06)
Real data
scCube (PCC$_{GEV}$ = 0.984 PCC$_{GBM}$ = 0.962)
SRTsim (PCC$_{GEV}$ = 0.983 PCC$_{GBM}$ = 0.967)
scDesign3 (PCC$_{GEV}$ = 0.027 PCC$_{GBM}$ = 0.206)
ZINB-WaVE (PCC$_{GEV}$ = 0.033 PCC$_{GBM}$ = -0.173)
SymSim (PCC$_{GEV}$ = -0.020 PCC$_{GBM}$ = -0.021)
scDesign2 (PCC$_{GEV}$ = -0.007 PCC$_{GBM}$ = 0.339)
Splat (PCC$_{GEV}$ = -0.008 PCC$_{GBM}$ = 0.135)
Kersplat (PCC$_{GEV}$ = 0.005 PCC$_{GBM}$ = -0.149)
Splat Simple (PCC$_{GEV}$ = -0.018 PCC$_{GBM}$ = -0.461)
Selplg (Microglia, Bregma: +0.06)
Real data
scCube (PCC$_{GEV}$ = 0.984 PCC$_{GBM}$ = 0.924)
SRTsim (PCC$_{GEV}$ = 0.960 PCC$_{GBM}$ = 0.864)
scDesign3 (PCC$_{GEV}$ = 0.030 PCC$_{GBM}$ = 0.143)
ZINB-WaVE (PCC$_{GEV}$ = 0.068 PCC$_{GBM}$ = 0.269)
SymSim (PCC$_{GEV}$ = -0.010 PCC$_{GBM}$ = 0.248)
scDesign2 (PCC$_{GEV}$ = -0.002 PCC$_{GBM}$ = -0.580)
Splat (PCC$_{GEV}$ = -0.014 PCC$_{GBM}$ = -0.207)
Kersplat (PCC$_{GEV}$ = 0.006 PCC$_{GBM}$ = -0.229)
Splat Simple (PCC$_{GEV}$ = 0.020 PCC$_{GBM}$ = -0.100)
Myh11 (Pericytes, Bregma: +0.06)
Real data
scCube (PCC$_{GEV}$ = 0.980 PCC$_{GBM}$ = 0.978)
SRTsim (PCC$_{GEV}$ = 0.970 PCC$_{GBM}$ = 0.979)
scDesign3 (PCC$_{GEV}$ = 0.186 PCC$_{GBM}$ = 0.844)
ZINB-WaVE (PCC$_{GEV}$ = 0.050 PCC$_{GBM}$ = 0.032)
SymSim (PCC$_{GEV}$ = -0.023 PCC$_{GBM}$ = 0.063)
scDesign2 (PCC$_{GEV}$ = -0.005 PCC$_{GBM}$ = -0.074)
Splat (PCC$_{GEV}$ = -0.012 PCC$_{GBM}$ = -0.065)
Kersplat (PCC$_{GEV}$ = -0.015 PCC$_{GBM}$ = 0.158)
Splat Simple (PCC$_{GEV}$ = 0.006 PCC$_{GBM}$ = 0.113)
Relative expression
0 0.2 0.4 0.6 0.8 1

Supplementary Figure S6. Performance comparison of scCube with other simulators over the mouse hypothalamus (Bregma: +0.06) dataset. The spatial expression patterns of nine representative cell type marker genes in the real data and the simulated data generated by scCube, SRTsim, scDesign3, ZINB-WaVE, SymSim, scDesign2, Splat, Kersplat, and Splat Simple. Source data are provided as a Source Data file.

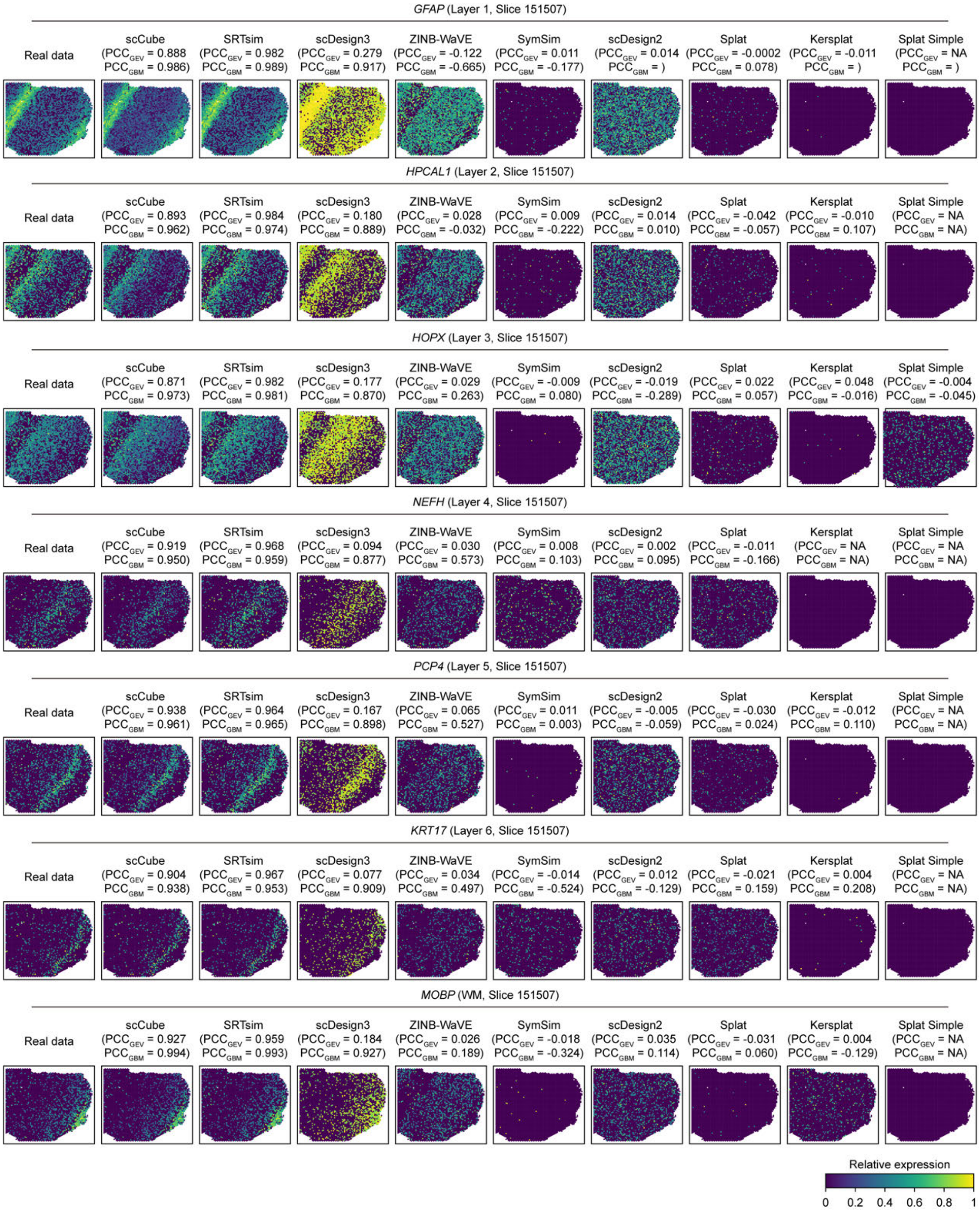

Supplementary Figure S7. Performance comparison of scCube with other simulators over the human DLPFC (Slice 151507) dataset. The spatial expression patterns of seven representative domain marker genes in the real data and the simulated data generated by scCube, SRTsim, scDesign3, ZINB-WaVE, SymSim, scDesign2, Splat, Kersplat, and Splat Simple. Source data are provided as a Source Data file.

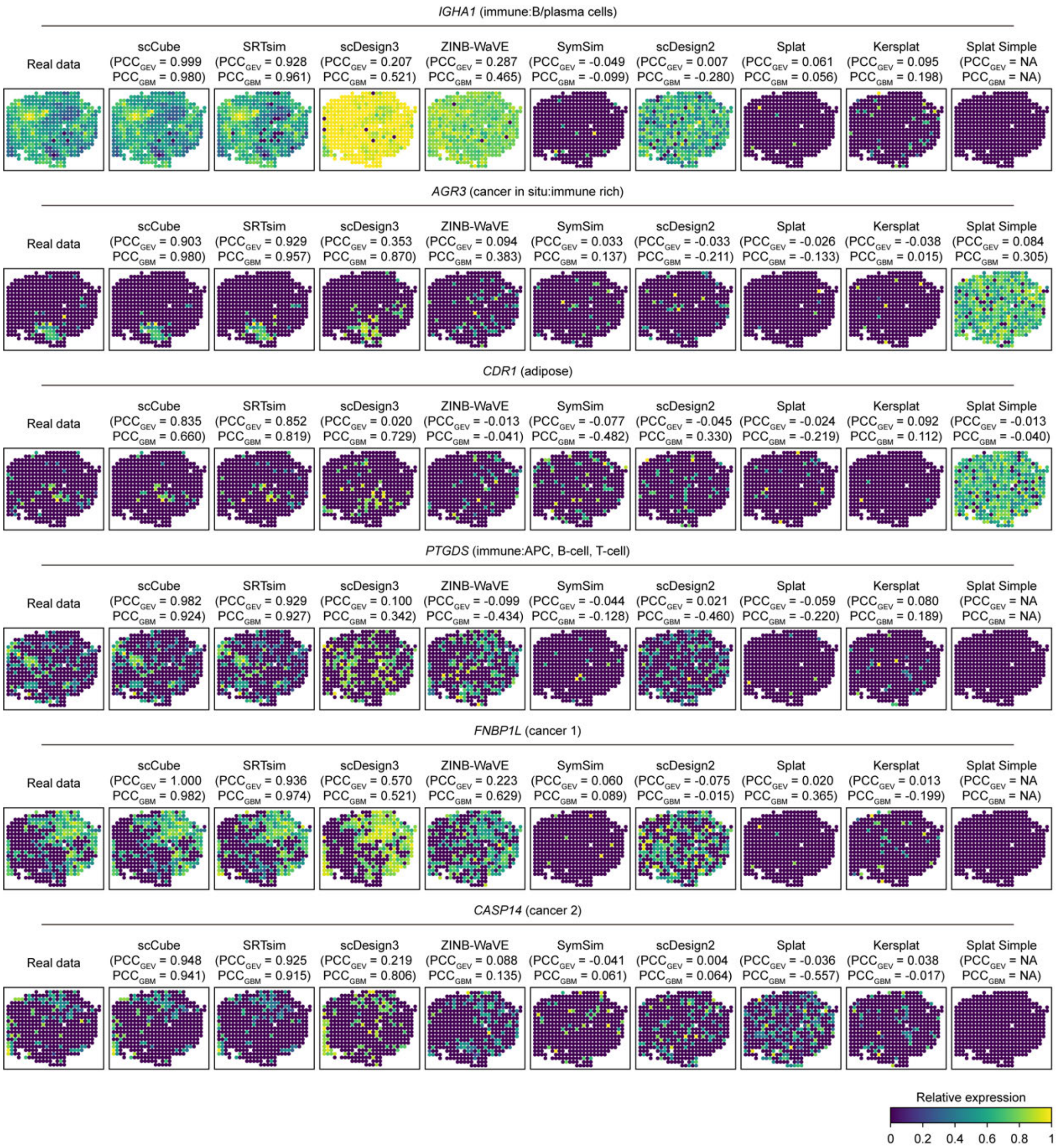

Supplementary Figure S8. Performance comparison of scCube with other simulators over the HER2-positive breast cancer (BC) ST (Patient G Replicate 1) dataset. The spatial expression patterns of six representative domain marker genes in the real data and the simulated data generated by scCube, SRTsim, scDesign3, ZINB-WaVE, SymSim, scDesign2, Splat, Kersplat, and Splat Simple. Source data are provided as a Source Data file.

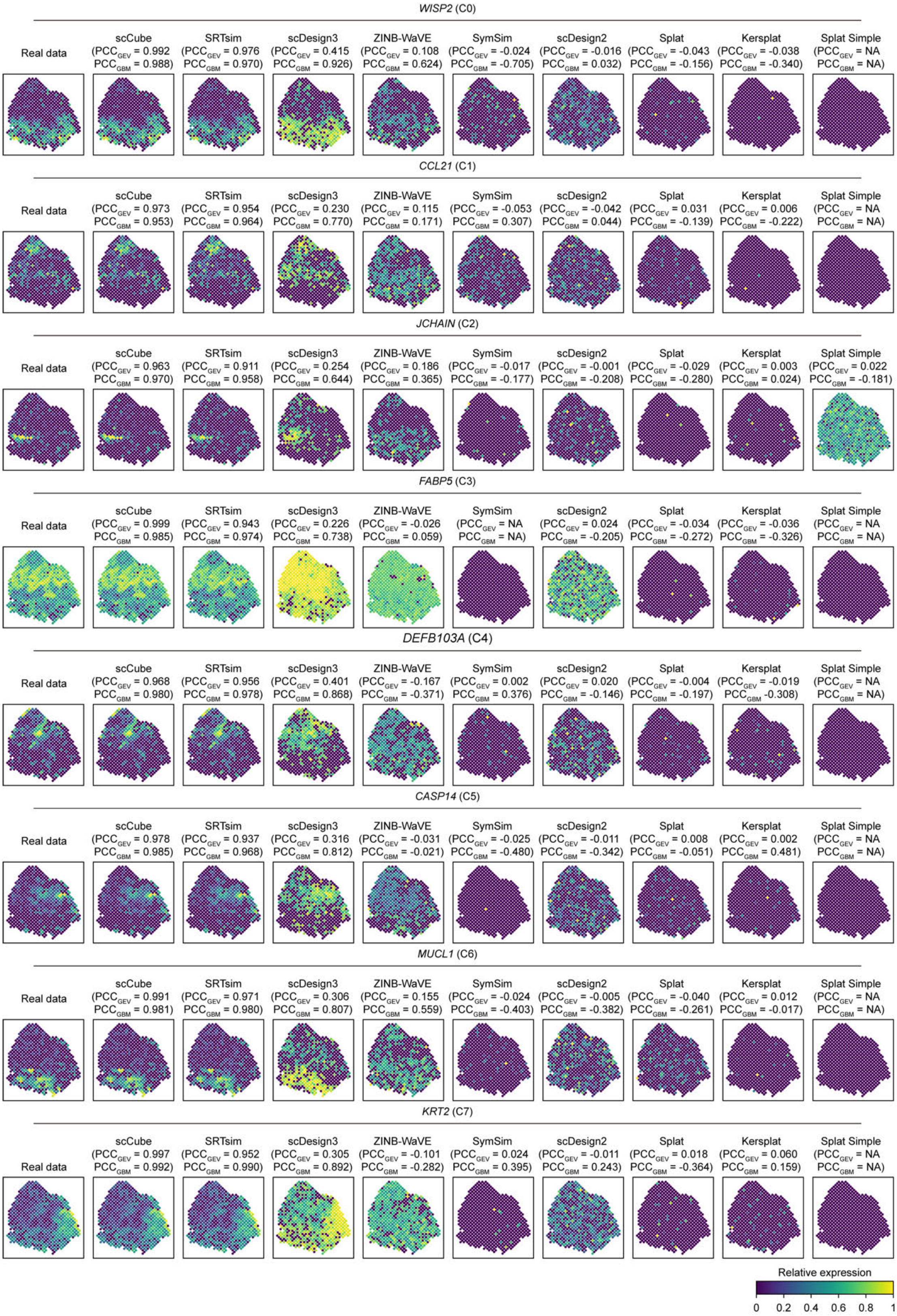

Supplementary Figure S9. Performance comparison of scCube with other simulators over the skin squamous cell carcinoma (SCC) ST (Patient 2 Replicate 1) dataset. The spatial expression patterns of eight representative domain marker genes in the real data and the simulated data generated by scCube, SRTsim, scDesign3, ZINB-WaVE, SymSim, scDesign2, Splat, Kersplat, and Splat Simple. Source data are provided as a Source Data file.

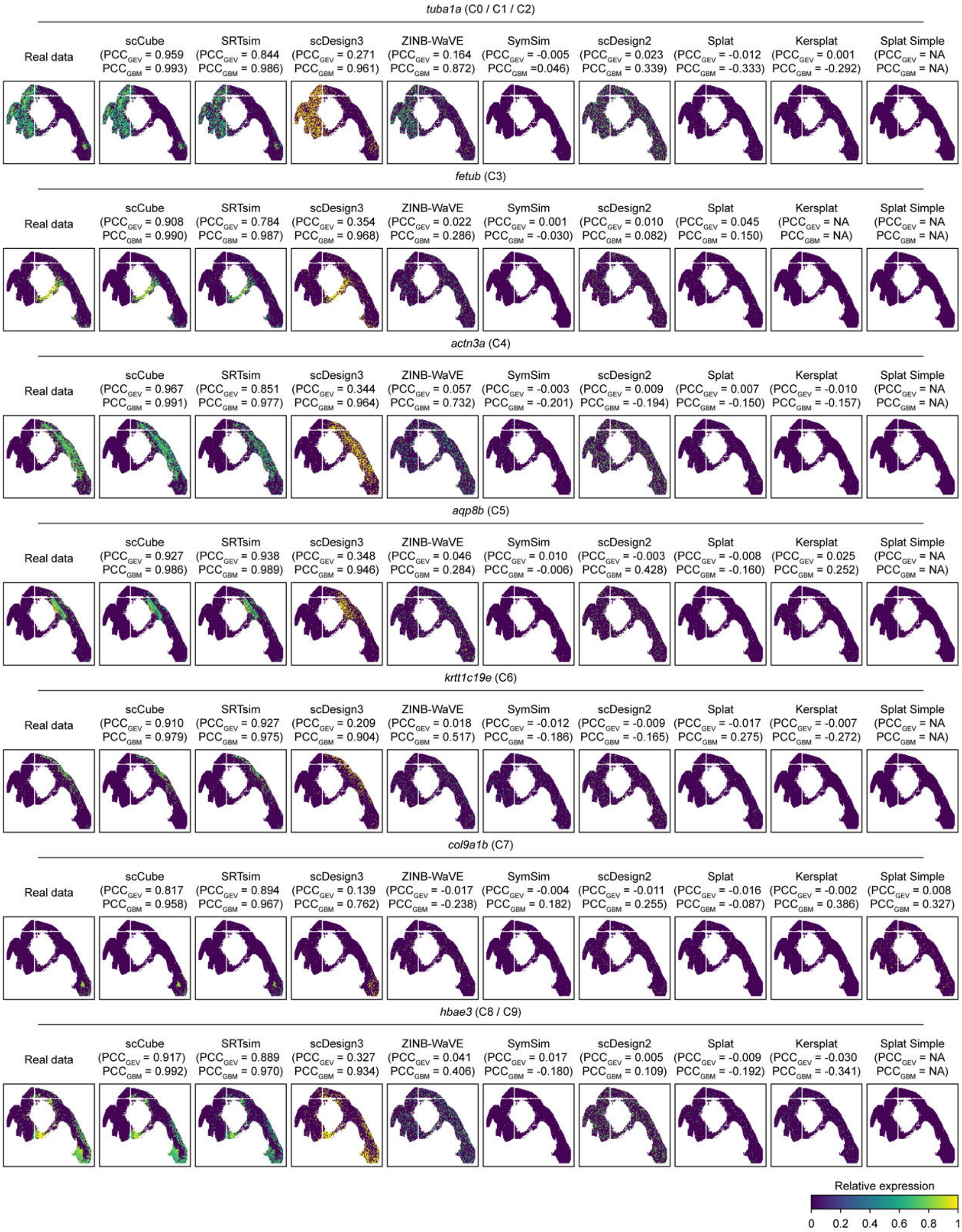

Supplementary Figure S10. Performance comparison of scCube with other simulators over the zebrafish embryo Stereo-seq (24-hpf) dataset. The spatial expression patterns of seven representative cluster marker genes in the real data and the simulated data generated by scCube, SRTsim, scDesign3, ZINB-WaVE, SymSim, scDesign2, Splat, Kersplat, and Splat Simple. Source data are provided as a Source Data file.

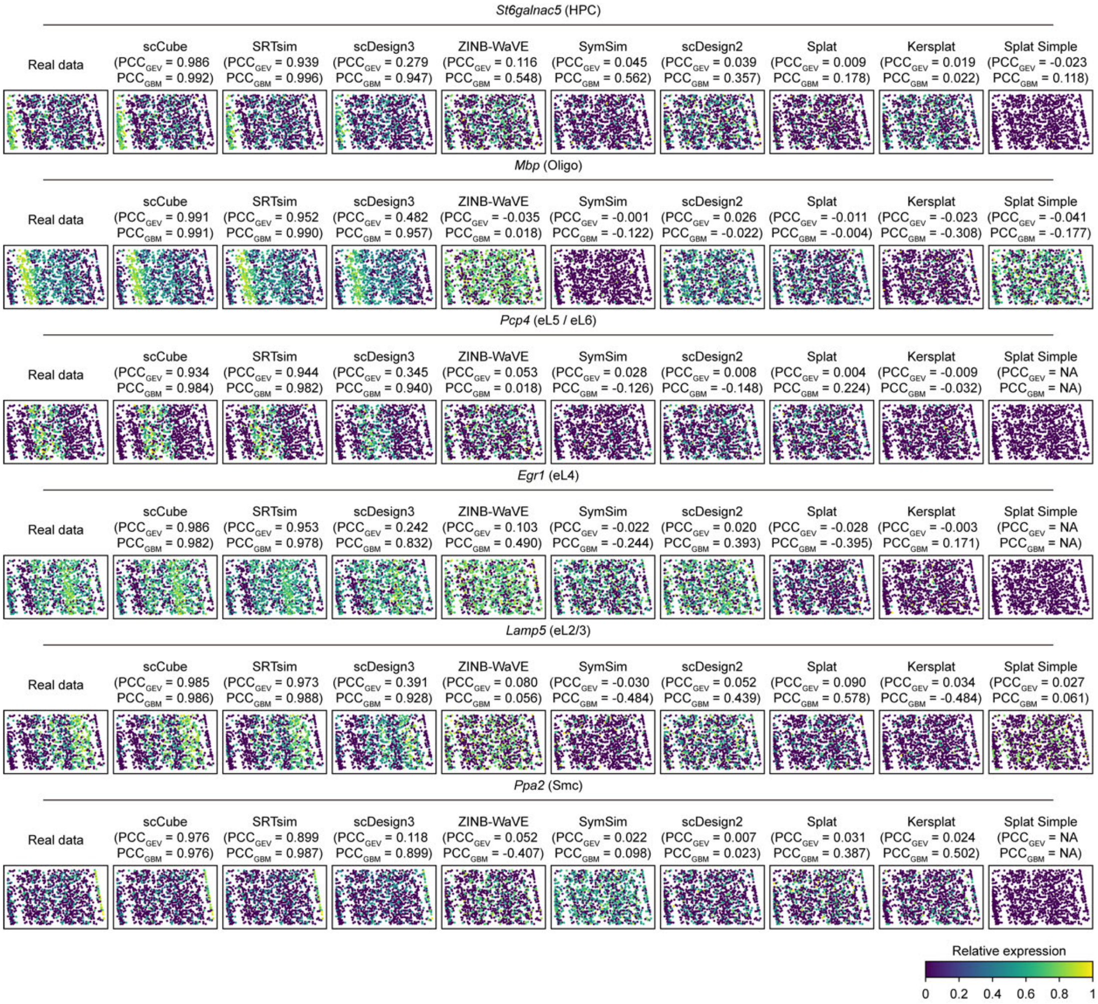

Supplementary Figure S11. Performance comparison of scCube with other simulators over the mouse V1 neocortex STARmap (Replicate 1) dataset. The spatial expression patterns of six representative cell type marker genes in the real data and the simulated data generated by scCube, SRTsim, scDesign3, ZINB-WaVE, SymSim, scDesign2, Splat, Kersplat, and Splat Simple. Source data are provided as a Source Data file.

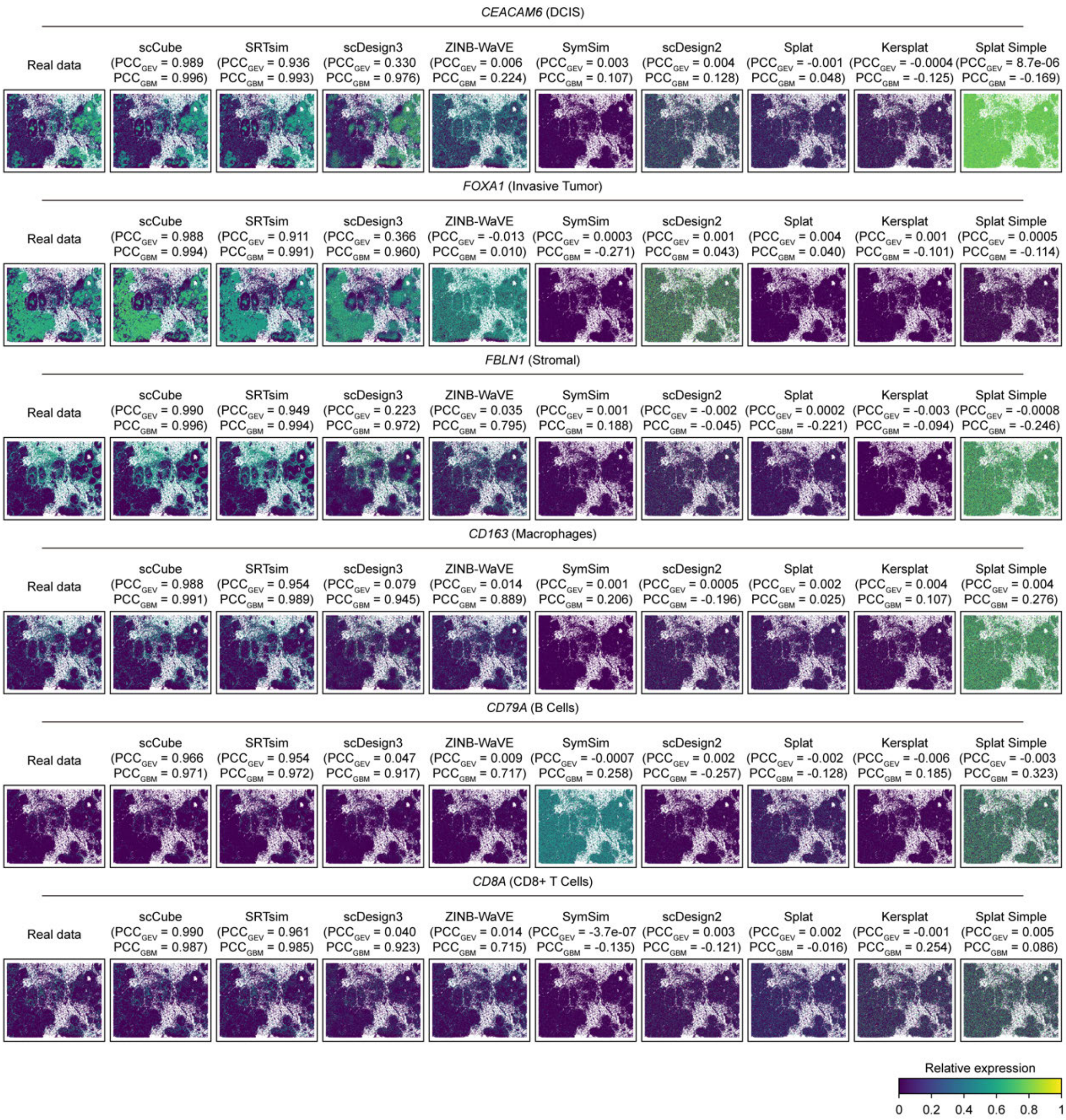

Supplementary Figure S12. Performance comparison of scCube with other simulators over the human breast cancer (BC) 10X Xenium (Sample 1 Replicate 1) dataset. The spatial expression patterns of seven representative cell type marker genes in the real data and the simulated data generated by scCube, SRTsim, scDesign3, ZINB-WaVE, SymSim, scDesign2, Splat, Kersplat, and Splat Simple. Source data are provided as a Source Data file.

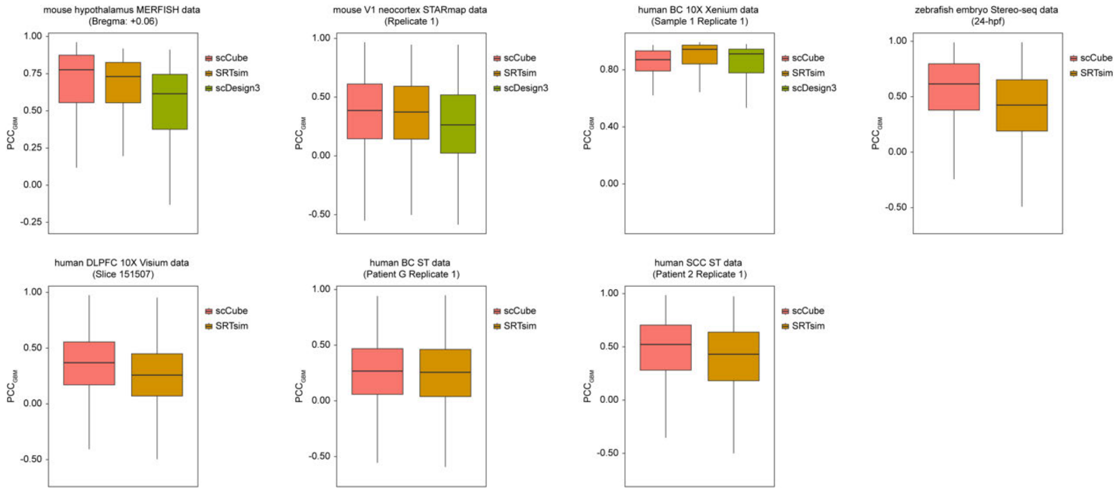

Supplementary Figure S13. Boxplots of PCC values between the two generalized boosted regression models' predicted gene expression values from the simulated data's spatial locations across seven benchmark datasets (scenario 1). The two generalized boosted regression models are trained on the real data and the simulated data generated by scCube and other two SRT simulators separately. The simulation results for all genes of scDesign3 are not provides in the Stereo-seq data, 10X Visium, and ST data due to speed constraints of the training step. Data are presented as boxplots (minima, 25th percentile, median, 75th percentile, and maxima). The number of data points are 155, 1,020, 313, 12,838, 18,094, 14,992, and 16,772 for mouse hypothalamus MERFISH data, mouse V1 neocortex STARmap data, human BC 10X Xenium data, zebrafish Stereo-seq data, human DLPFC 10X Visium data, human BC ST data, and human SCC ST data, respectively. Source data are provided as a Source Data file.

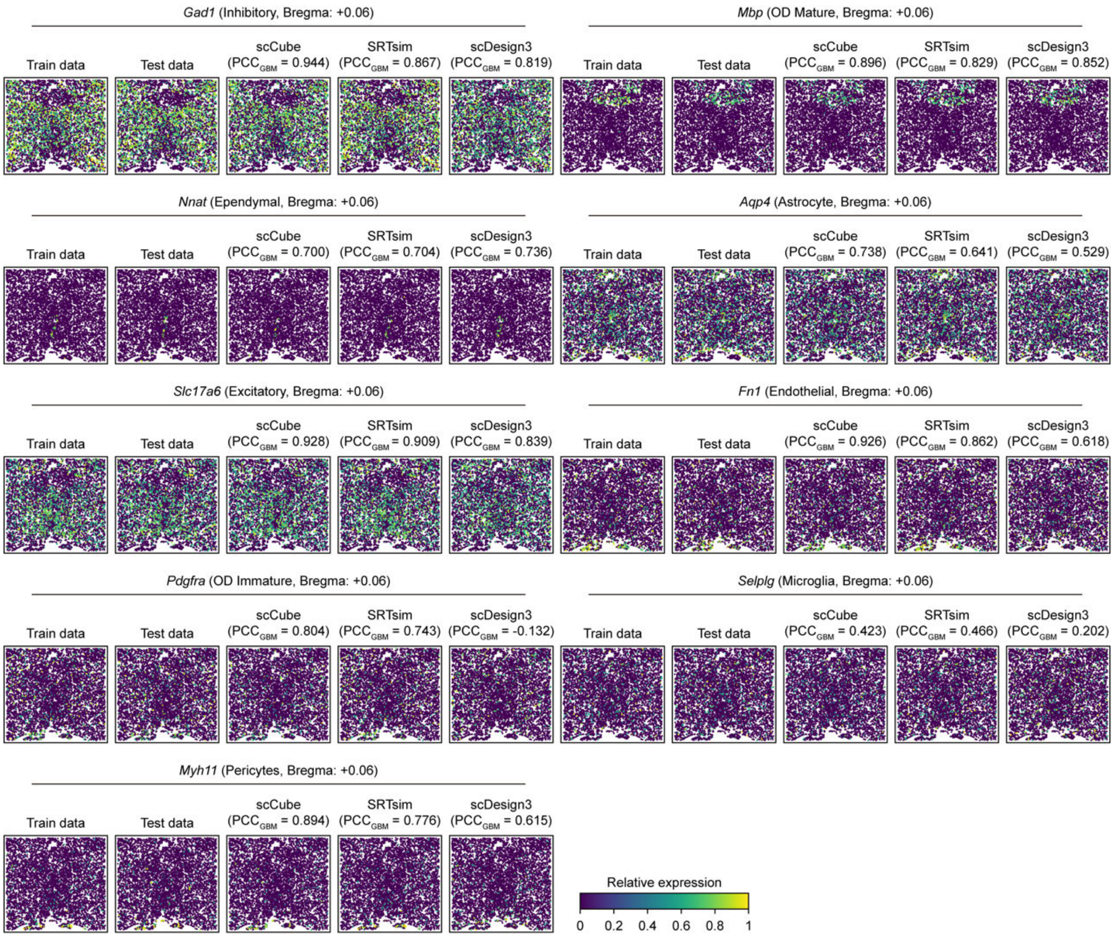

Supplementary Figure S14. Performance comparison of scCube with other two SRT simulators over the mouse hypothalamus MERFISH (Bregma: +0.06) dataset split by countsplit. Source data are provided as a Source Data file.

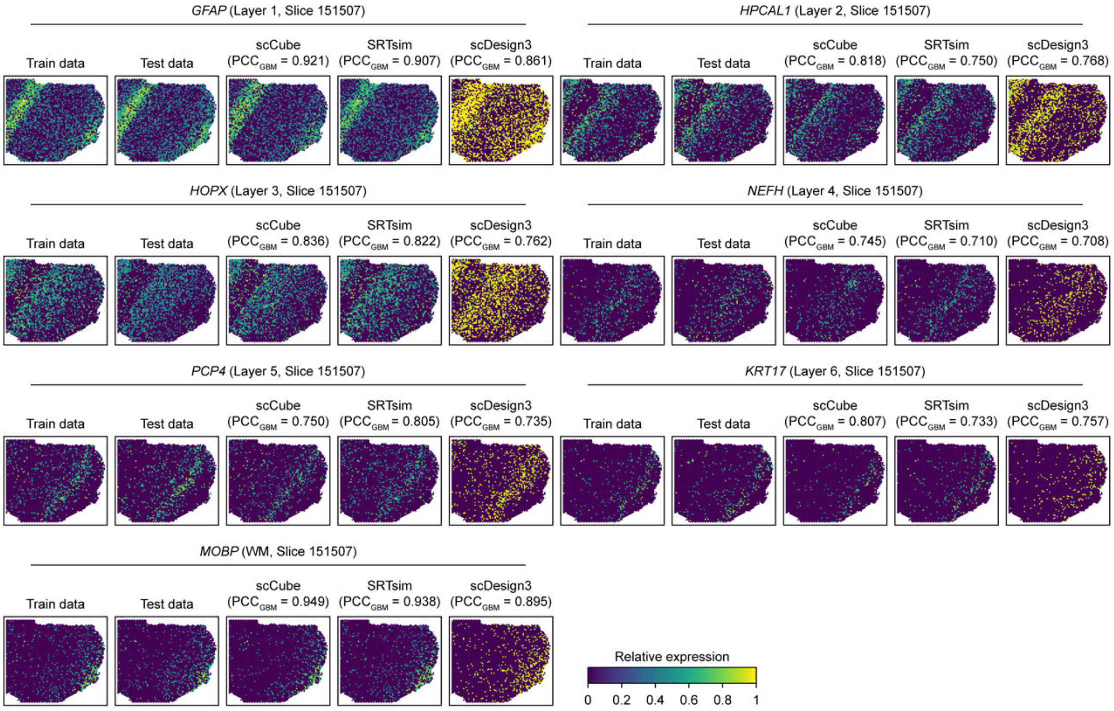

Supplementary Figure S15. Performance comparison of scCube with other two SRT simulators over the human DLPFC 10X Visium (Slice 151507) dataset split by countsplit. Source data are provided as a Source Data file.

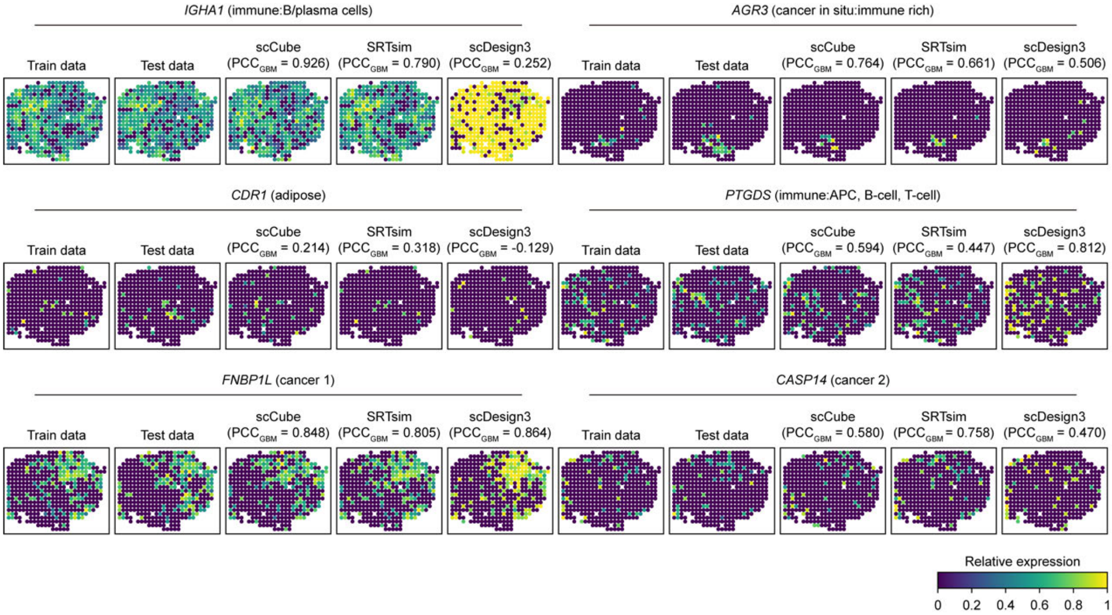

Supplementary Figure S16. Performance comparison of scCube with other two SRT simulators over the HER2-positive breast cancer (BC) ST (Patient G Replicate 1) dataset split by countsplit. Source data are provided as a Source Data file.

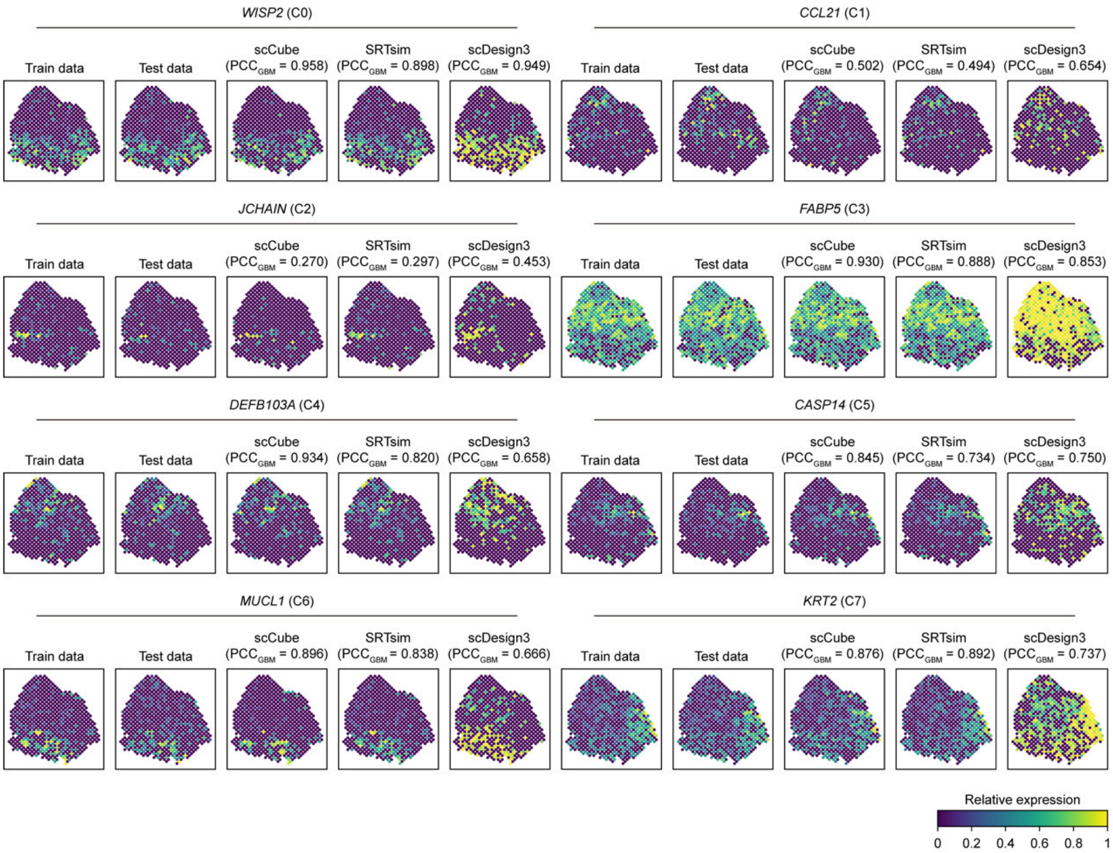

Supplementary Figure S17. Performance comparison of scCube with other two SRT simulators over the skin squamous cell carcinoma (SCC) ST (Patient 2 Replicate 1) dataset split by countsplit. Source data are provided as a Source Data file.

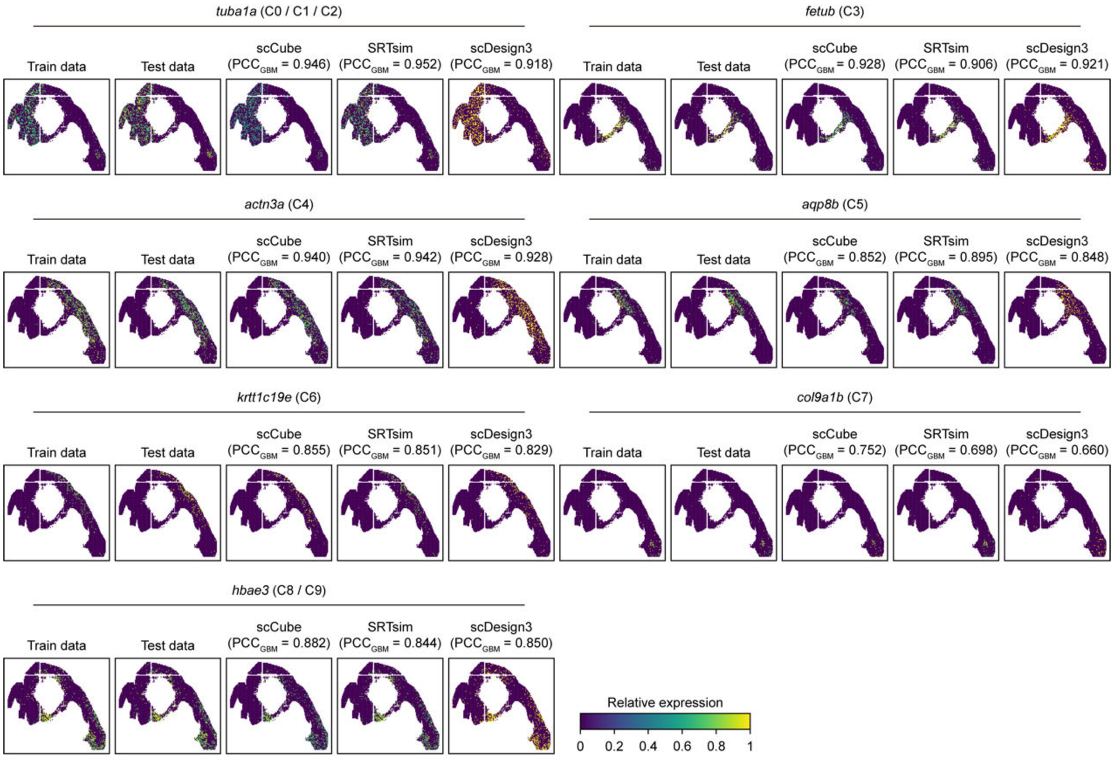

Supplementary Figure S18. Performance comparison of scCube with other two SRT simulators over the zebrafish embryo Stereo-seq (24-hpf) dataset split by countsplit. Source data are provided as a Source Data file.

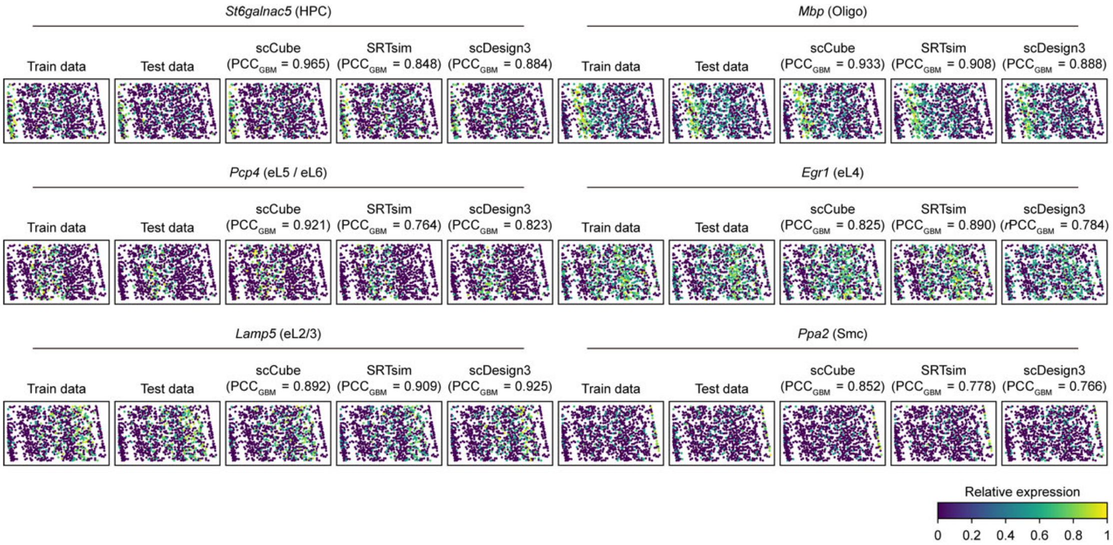

Supplementary Figure S19. Performance comparison of scCube with other two SRT simulators over the mouse V1 neocortex STARmap (Replicate 1) dataset split by countsplit. Source data are provided as a Source Data file.

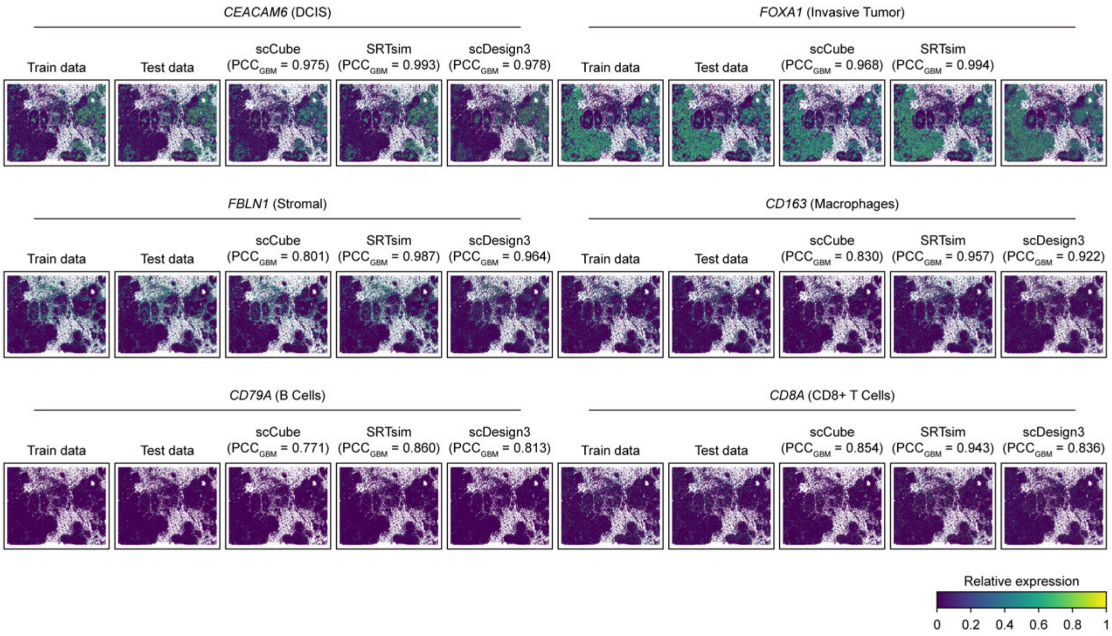

Supplementary Figure S20. Performance comparison of scCube with other two SRT simulators over the human breast cancer (BC) 10X Xenium (Sample 1 Replicate 1) dataset split by countsplit. Source data are provided as a Source Data file.

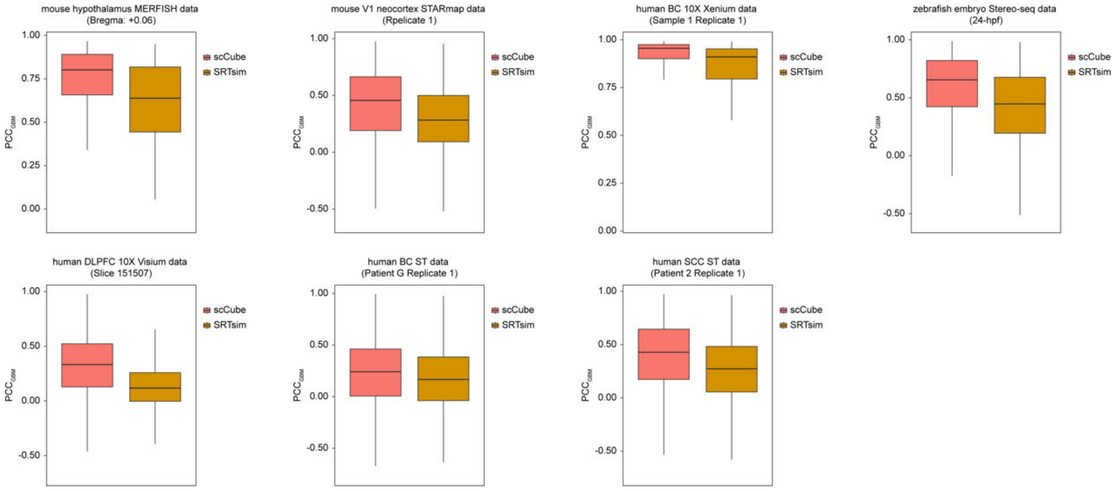

Supplementary Figure S21. Boxplots of PCC values between the two generalized boosted regression models' predicted gene expression values from the simulated data's spatial locations across seven benchmark datasets (scenario 2). The two generalized boosted regression models are trained on the real data and the simulated data generated by scCube and SRTsim separately. Data are presented as boxplots (minima, 25th percentile, median, 75th percentile, and maxima). The number of data points are 155, 1,020, 313, 12,838, 18,094, 14,992, and 16,772 for mouse hypothalamus MERFISH data, mouse V1 neocortex STARmap data, human BC 10X Xenium data, zebrafish Stereo-seq data, human DLPFC 10X Visium data, human BC ST data, and human SCC ST data, respectively. Source data are provided as a Source Data file.

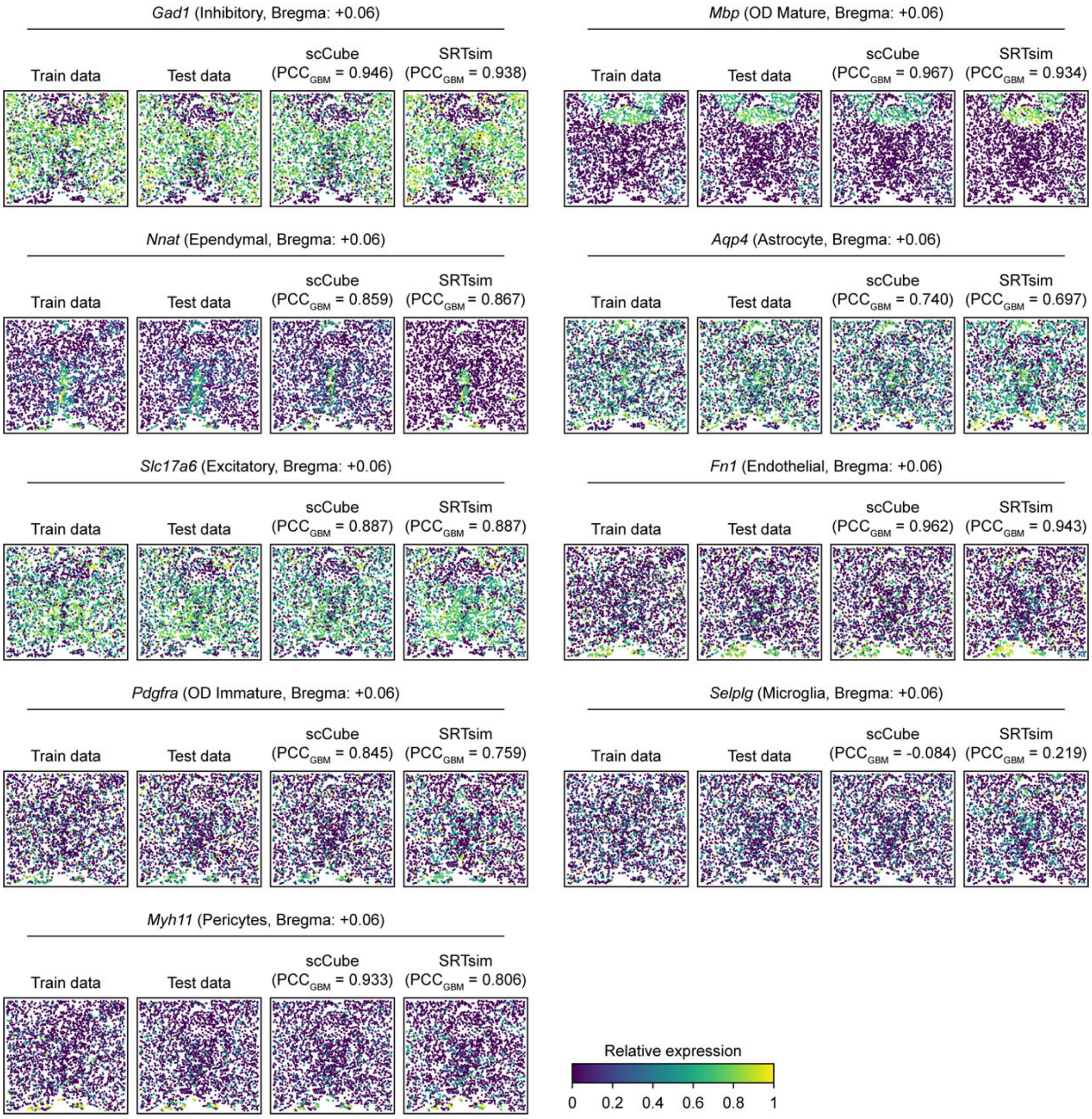

Supplementary Figure S22. Performance comparison of scCube with other two SRT simulators over the mouse hypothalamus MERFISH (Bregma: +0.06) dataset split by the random splitting strategy. Source data are provided as a Source Data file.

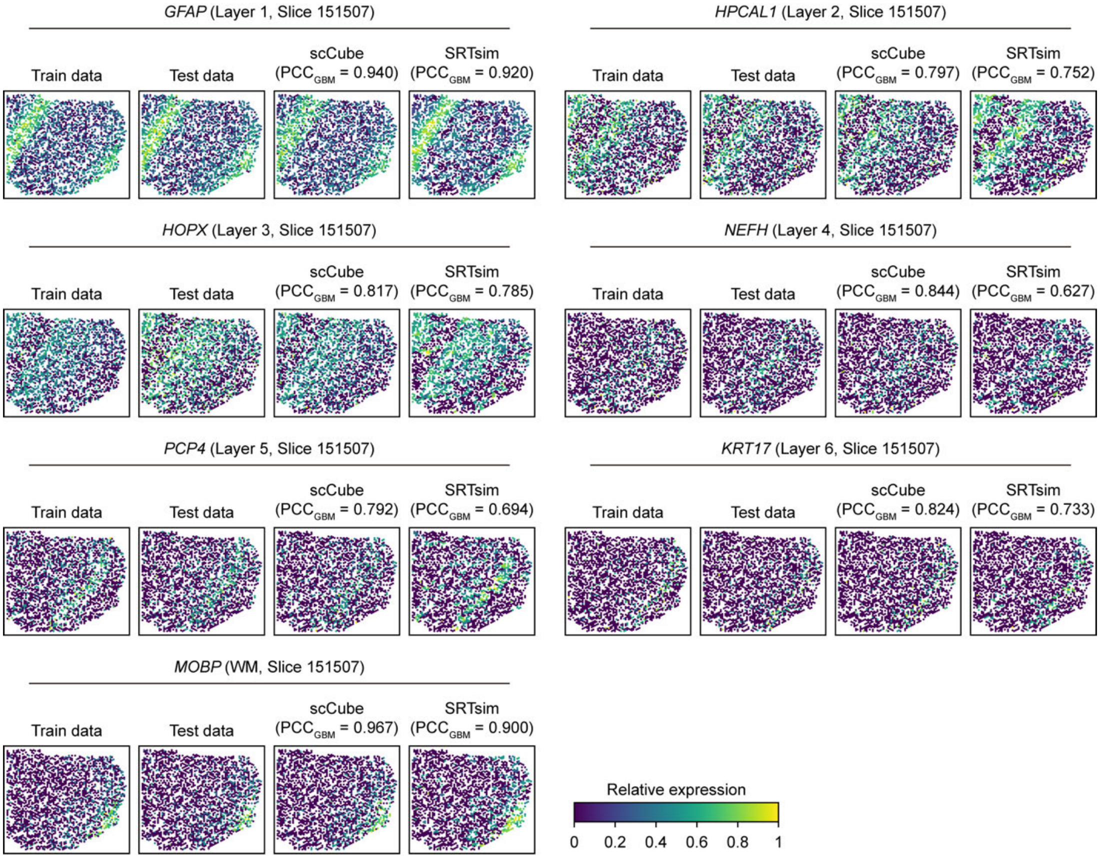

Supplementary Figure S23. Performance comparison of scCube with other two SRT simulators over the human DLPFC 10X Visium (Slice 151507) dataset split by the random splitting strategy. Source data are provided as a Source Data file.

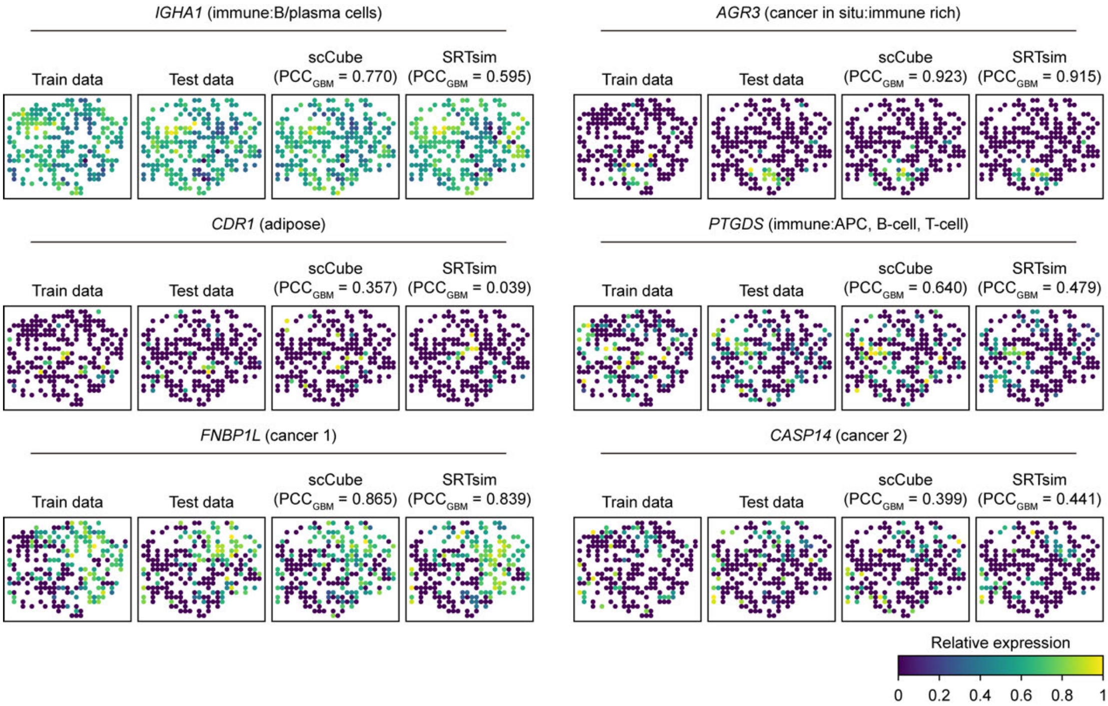

Supplementary Figure S24. Performance comparison of scCube with other two SRT simulators over the HER2-positive breast cancer (BC) ST (Patient G Replicate 1) dataset split by the random splitting strategy. Source data are provided as a Source Data file.

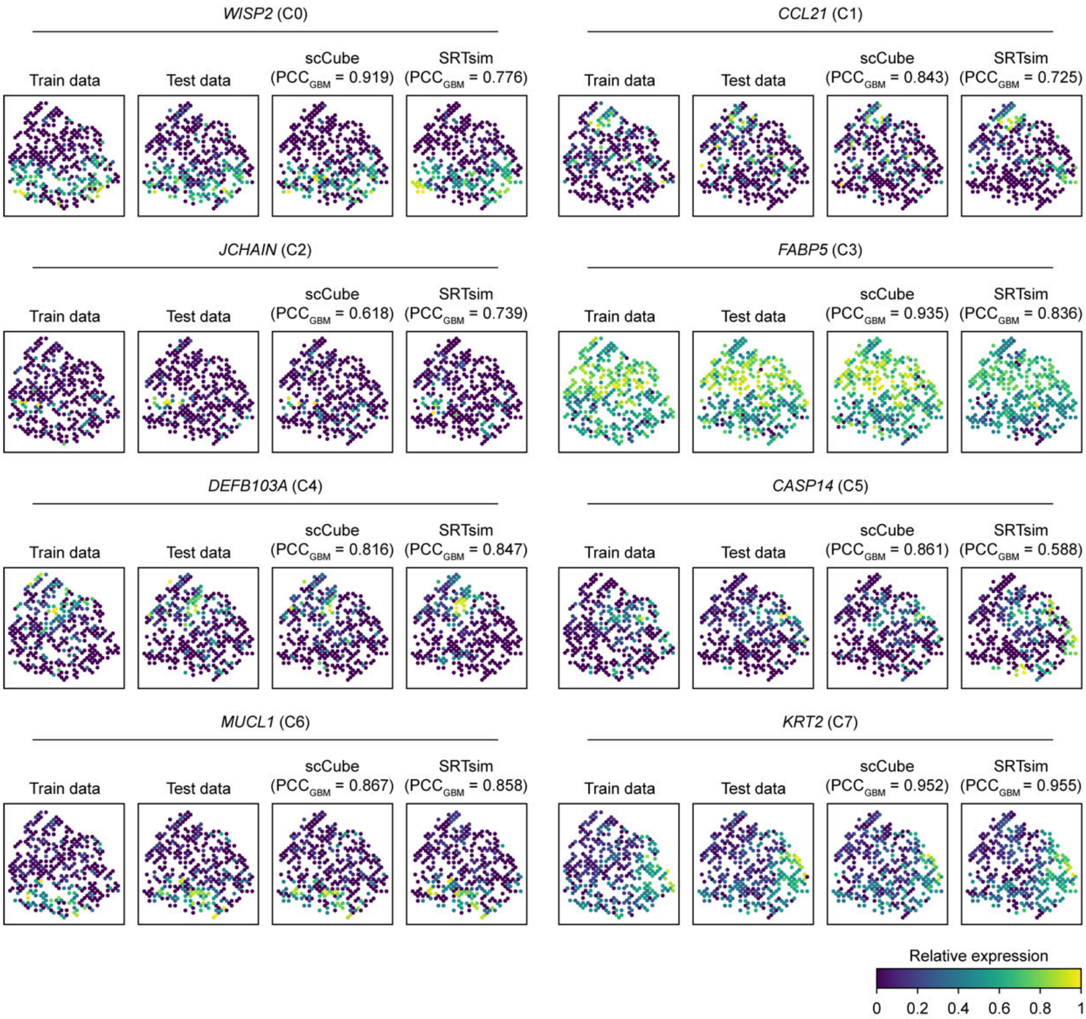

Supplementary Figure S25. Performance comparison of scCube with other two SRT simulators over the skin squamous cell carcinoma (SCC) ST (Patient 2 Replicate 1) dataset split by the random splitting strategy. Source data are provided as a Source Data file.

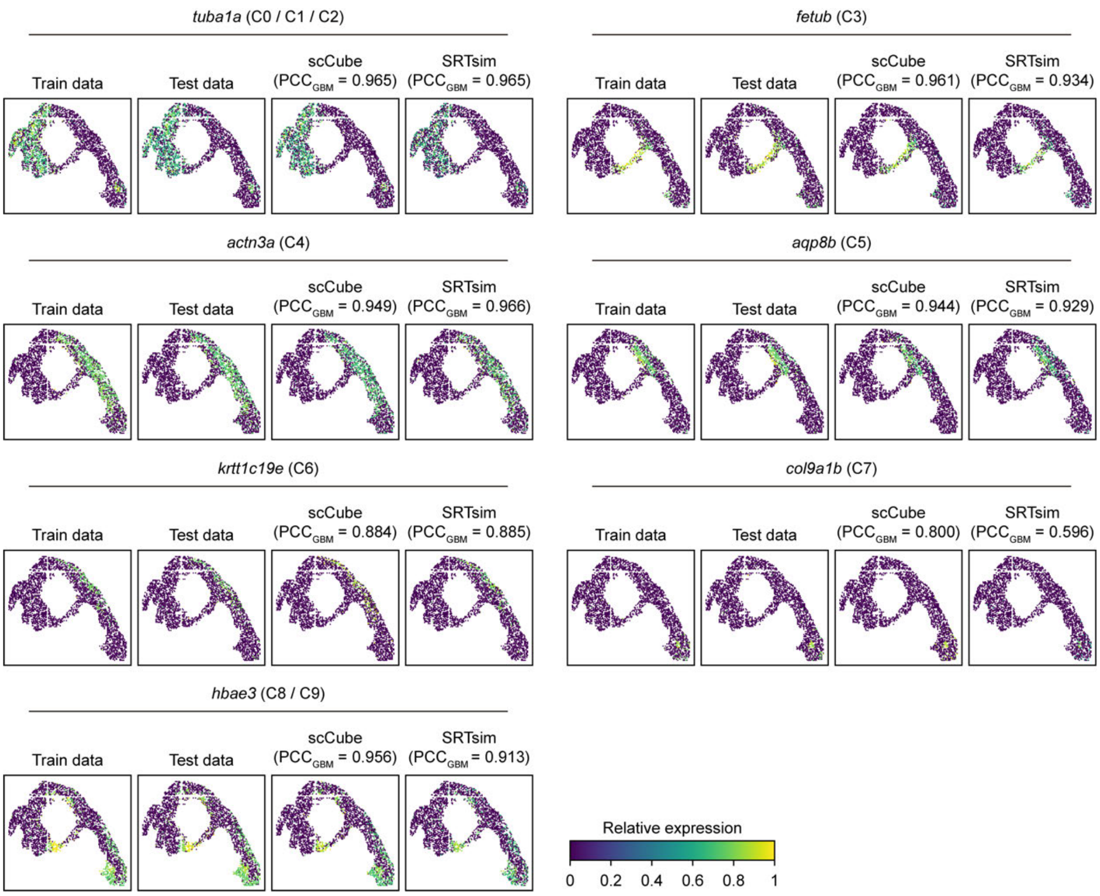

Supplementary Figure S26. Performance comparison of scCube with other two SRT simulators over the zebrafish embryo Stereo-seq (24-hpf) dataset split by the random splitting strategy. Source data are provided as a Source Data file.

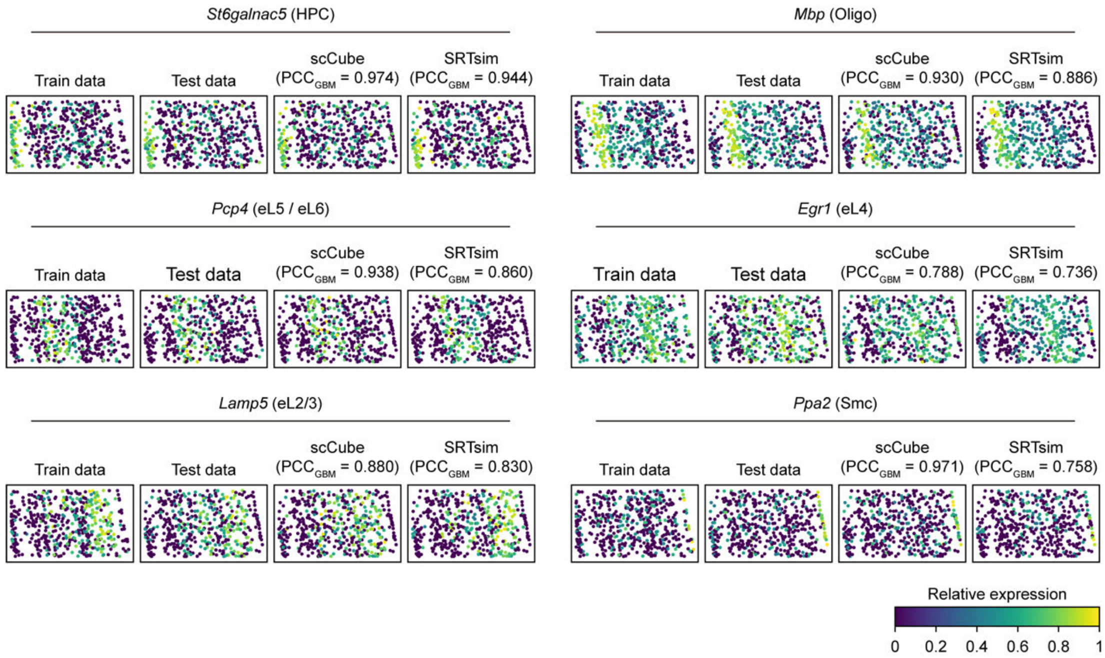

Supplementary Figure S27. Performance comparison of scCube with other two SRT simulators over the mouse V1 neocortex STARmap (Replicate 1) dataset split by the random splitting strategy. Source data are provided as a Source Data file.

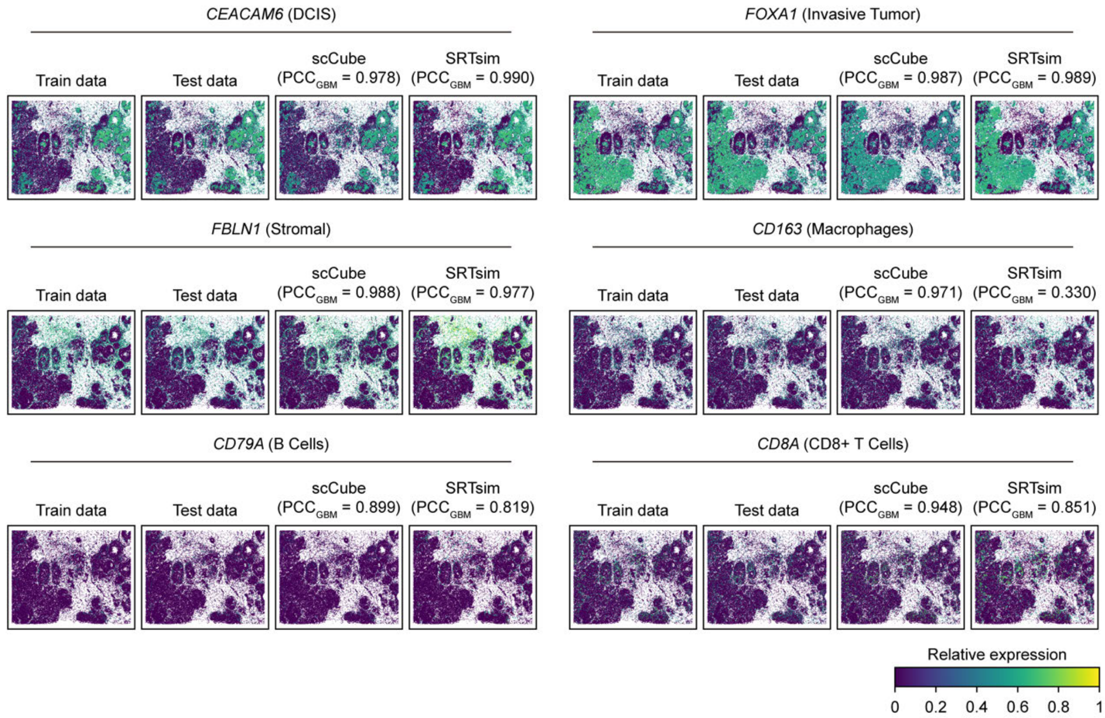

Supplementary Figure S28. Performance comparison of scCube with other two SRT simulators over the human breast cancer (BC) 10X Xenium (Sample 1 Replicate 1) dataset split by the random splitting strategy. Source data are provided as a Source Data file.

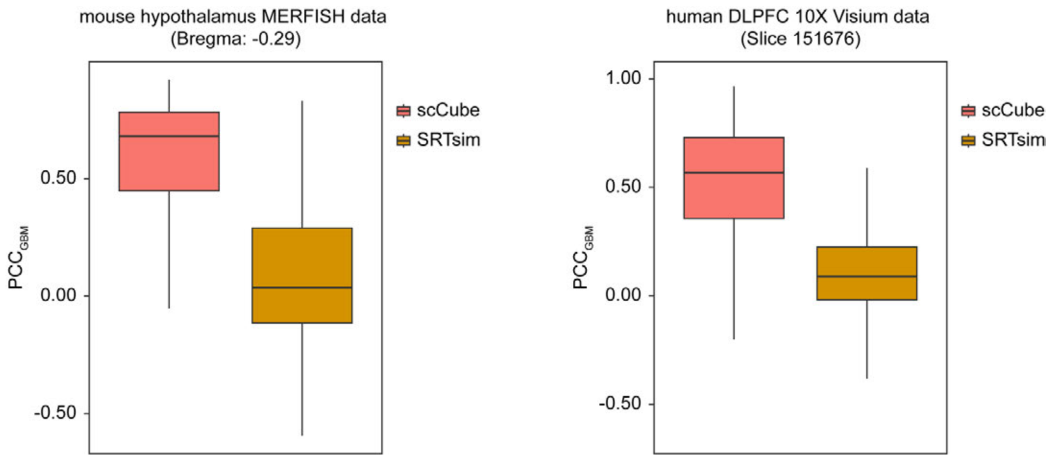

Supplementary Figure S29. Boxplots of PCC values between the two generalized boosted regression models' predicted gene expression values from the simulated data's spatial locations across two benchmark datasets (scenario 3). The two generalized boosted regression models are trained on the real data and the simulated data generated by scCube and SRTsim separately. Data are presented as boxplots (minima, 25th percentile, median, 75th percentile, and maxima). The number of data points are 155 and 18,094 for mouse hypothalamus MERFISH data and human DLPFC 10X Visium data, respectively. Source data are provided as a Source Data file.

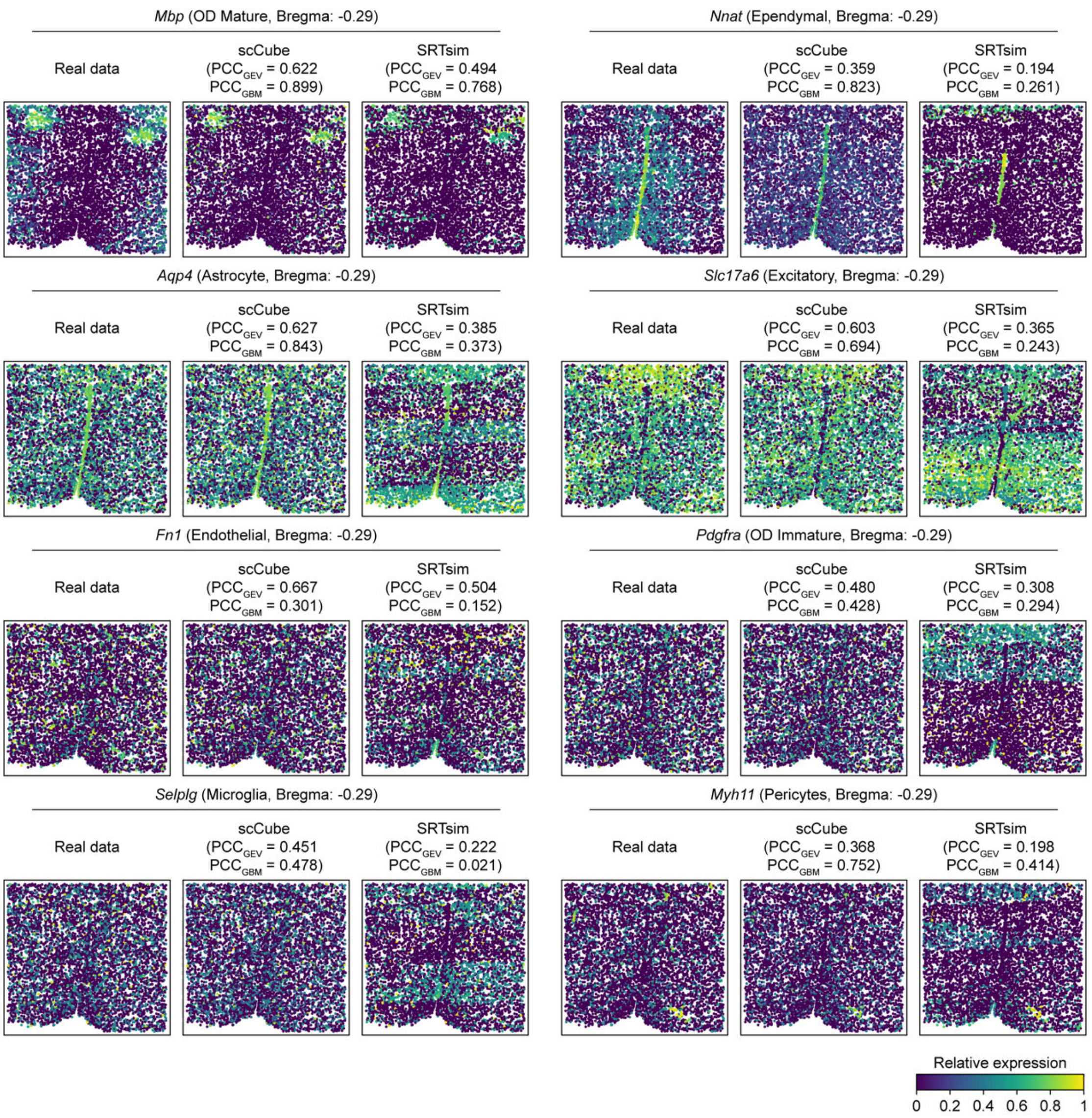

Supplementary Figure S30. Performance comparison of scCube with SRTsim over the mouse hypothalamus MERFISH (Bregma: -0.29) dataset using the mouse hypothalamus MERFISH (Bregma: +0.06) dataset as the spatial reference. The spatial expression patterns of other eight cell type marker genes in the real data and the simulated data generated by scCube and SRTsim. Source data are provided as a Source Data file.

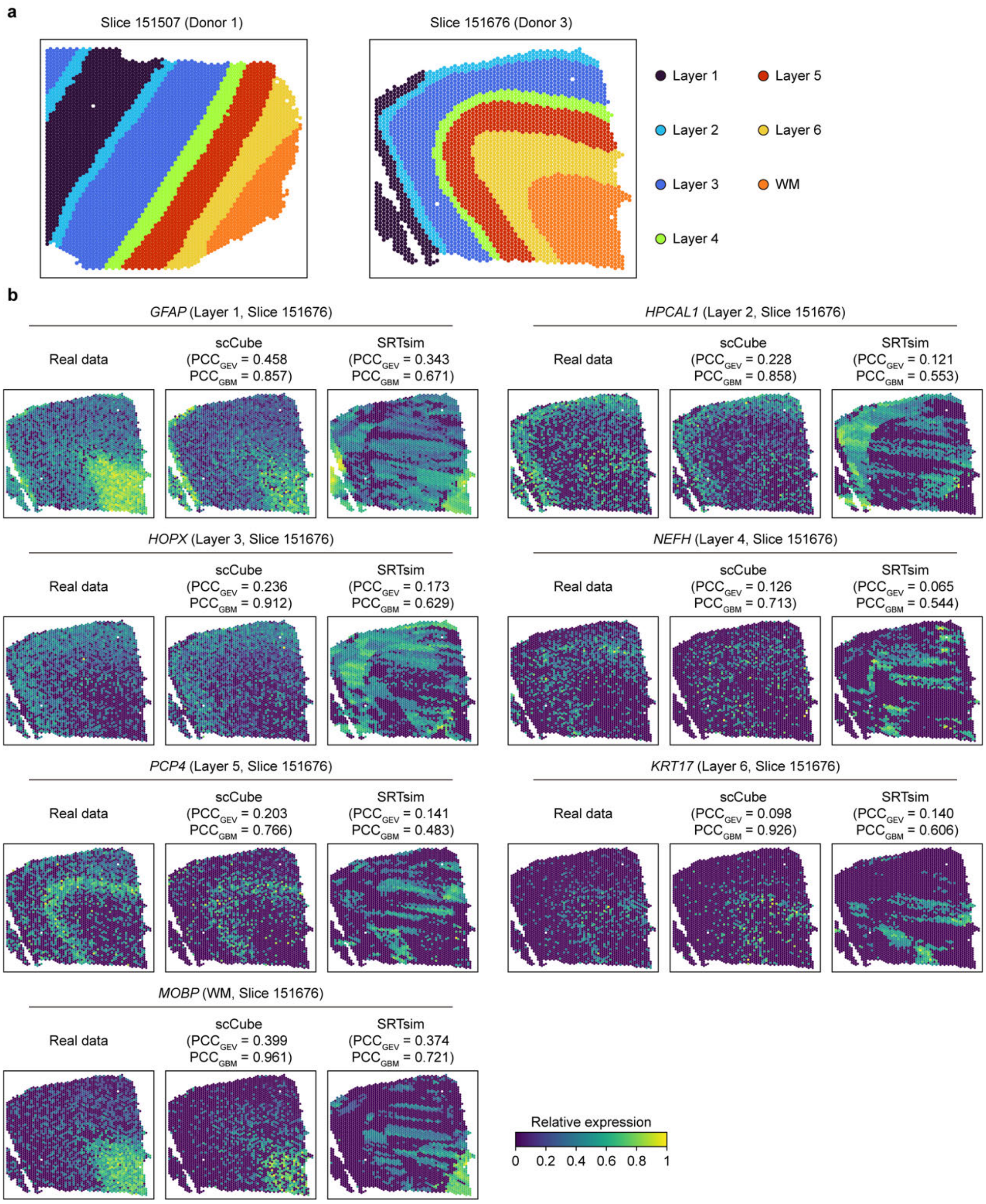

Supplementary Figure S31. Performance comparison of scCube with SRTsim over the human DLPFC 10X Visium (Slice 151676) dataset using the human DLPFC 10X Visium (Slice 151507) dataset as the spatial reference. a, Two tissue slices with different shapes from the human DLPFC dataset, where the Slice 151507 is from donor 1 and the Slice 151676 is from donor 3. **b,** The spatial expression patterns of seven domain marker genes in the real data and the simulated data generated by scCube and SRTsim. Source data are provided as a Source Data file.

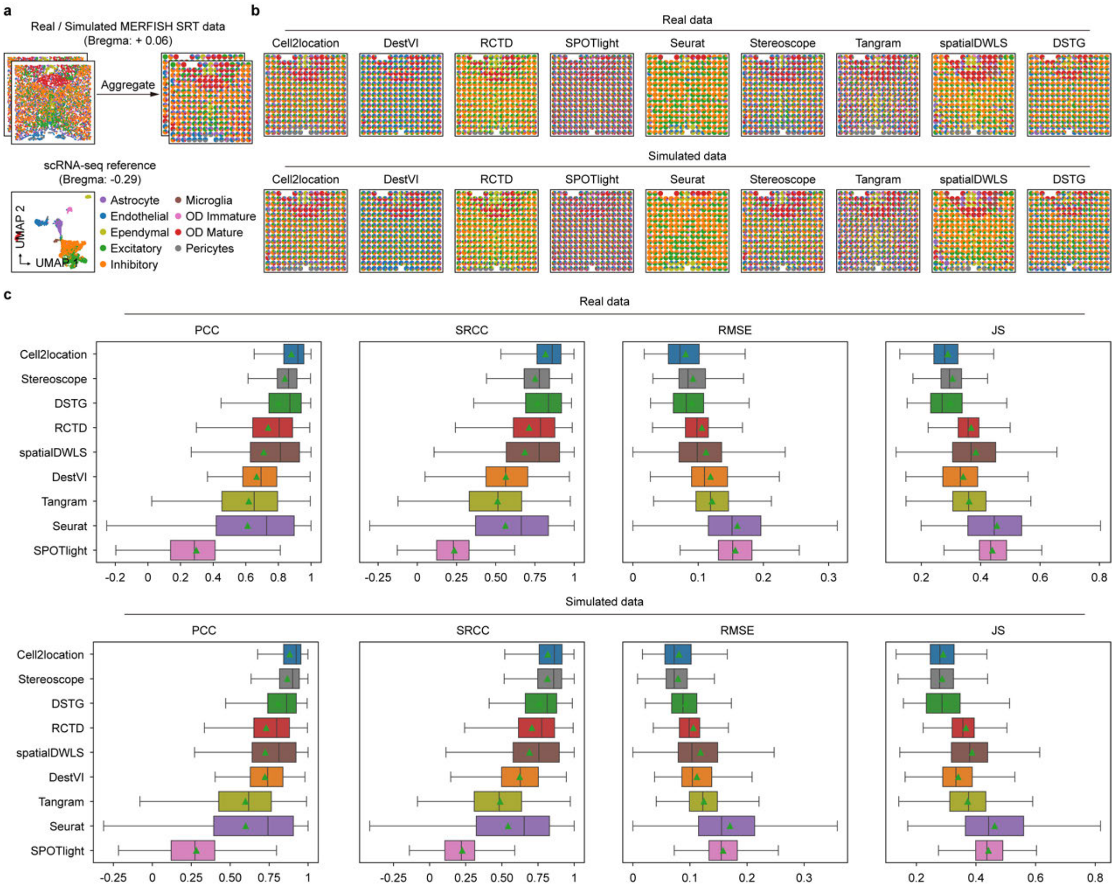

Supplementary Figure S32. Comparison of benchmark results of spot deconvolution methods between the real and scCube-simulated data. a, Schematic of benchmarking spot deconvolution methods. The real and scCube-simulated mouse hypothalamus MERFISH data (Bregma: + 0.06) are aggregated respectively as the SRT data to be deconvolved, and the mouse hypothalamus MERFISH data from another slice (Bregma: - 0.29) is utilized as the single-cell reference. **b**, The deconvolution results of each method over the real (top) and scCube-simulated (bottom) SRT data. **c**, The benchmark results of all methods over the real (top) and scCube-simulated (bottom) SRT data. Data are presented as boxplots (minima, 25th percentile, median, 75th percentile, and maxima). The number of data points are 222 for each method. Source data are provided as a Source Data file.

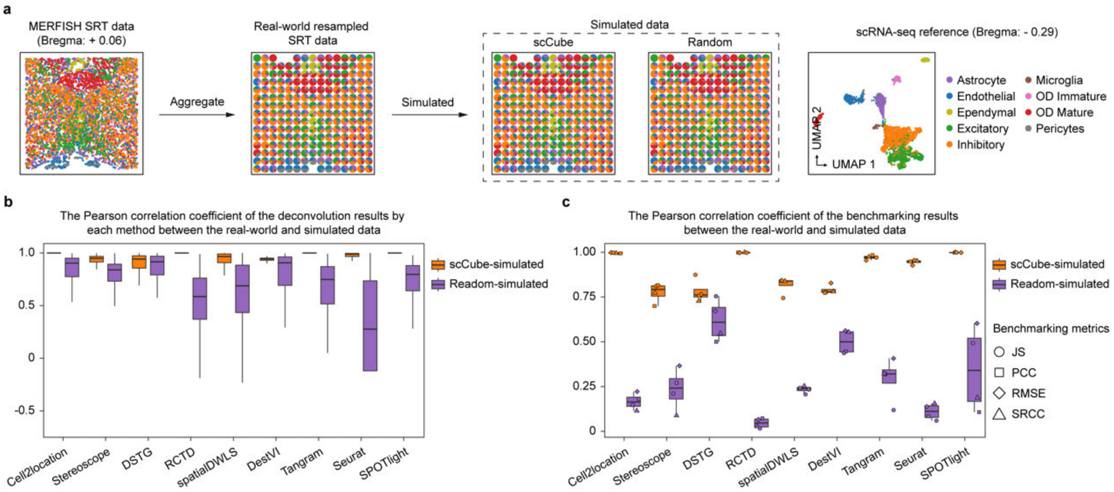

Supplementary Figure S33. Comparison of benchmark results of spot deconvolution methods between the real, scCube-simulated, and random-simulated data. a, Schematic of benchmarking spot deconvolution methods. The real-world resampled SRT dataset is aggregated from the MERFISH data (Bregma: +0.06). Next, based on this SRT dataset, two simulated SRT datasets are generated by scCube and random strategies, respectively. The random-simulated dataset is generated by combining corresponding number and types of cells randomly selected from the MERFISH (Bregma: +0.06) data according to the ground truth of the composition of cells in each spot. The MERFISH data from another slice (Bregma: -0.29) is utilized as the scRNA-seq reference. **b,** The comparison of the deconvolution results of each spot by each method between the real-world and simulated data. Data are presented as boxplots (minima, 25th percentile, median, 75th percentile, and maxima). The number of data points are 222 for each method. **c,** The comparison of the benchmarking results of each method between the real-world and simulated data. Four different benchmarking metrics were utilized. Data are presented as boxplots (minima, 25th percentile, median, 75th percentile, and maxima). The number of data points are 4 for each method. Source data are provided as a Source Data file.

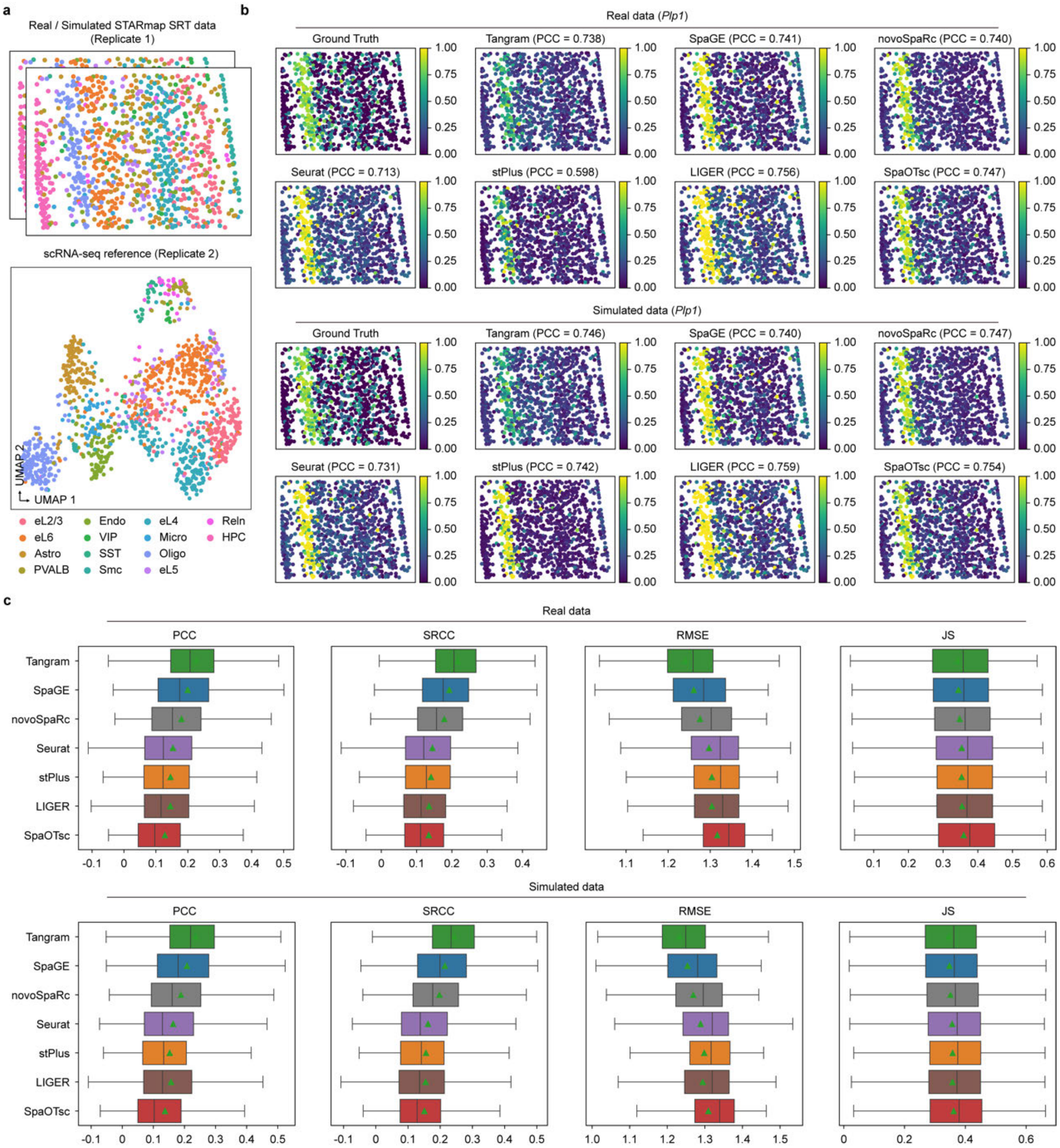

Supplementary Figure S34. Comparison of benchmark results of gene imputation methods between the real and scCube-simulated data. a, Schematic of benchmarking gene imputation methods. The real and scCube-simulated mouse V1 neocortex STARmap data (Replicate 1) are selected as the SRT data to be imputed, and the mouse V1 neocortex STARmap data from another slice (Replicate 2) is utilized as the single-cell reference. **b**, The gene imputation results of each method over the real (top) and scCube-simulated (bottom) SRT data. **c**, The benchmark results of all methods over the real (top) and scCube-simulated (bottom) SRT data. Data are presented as boxplots (minima, 25th percentile, median, 75th percentile, and maxima). The number of data points are 1,020 for each method. Source data are provided as a Source Data file.

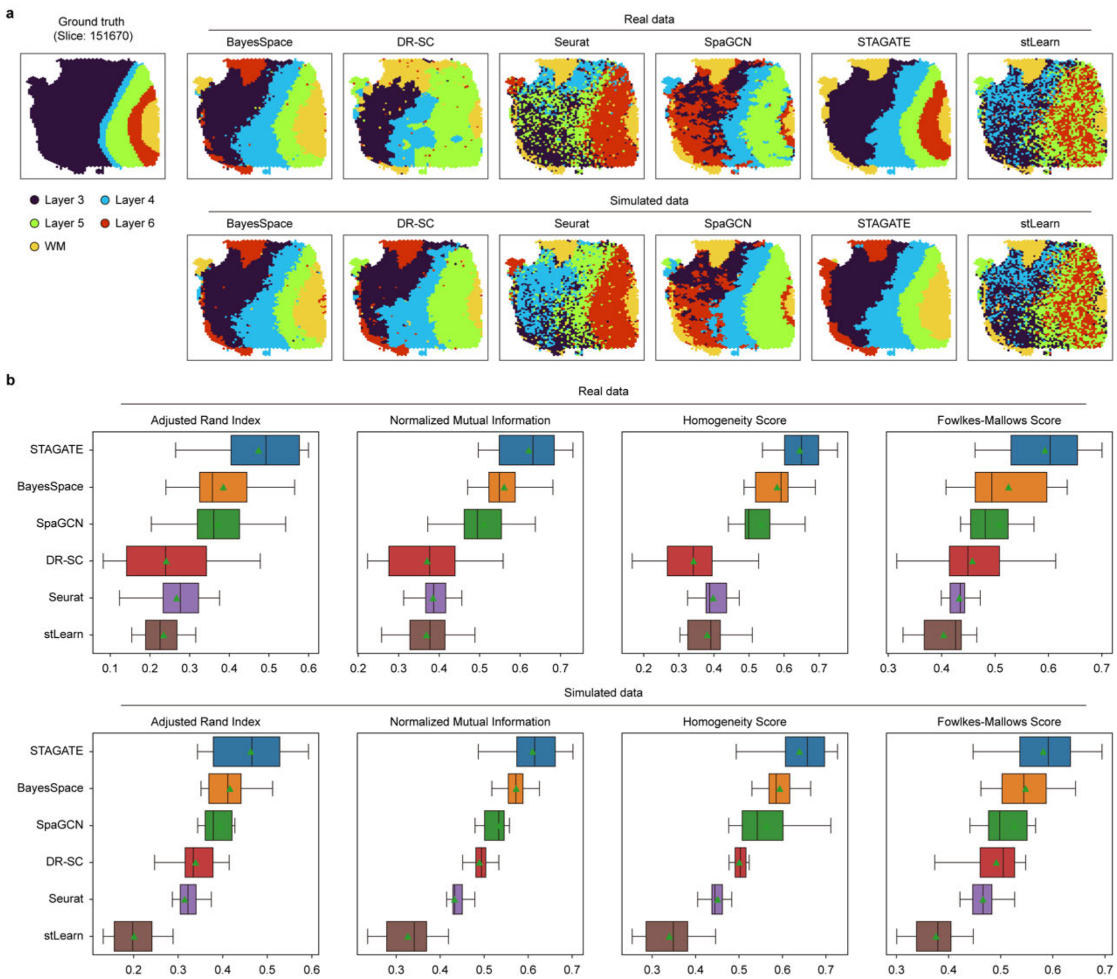

Supplementary Figure S35. Comparison of benchmark results of spatial domain identification methods between the real and scCube-simulated data. a, The clustering results of each method over the real (top) and scCube-simulated (bottom) SRT data (human DLPFC 10X Visium data, Slice: 151670). **b**, The benchmark results of all methods over the real (top) and scCube-simulated (bottom) SRT data. Data are presented as boxplots (minima, 25th percentile, median, 75th percentile, and maxima). The number of data points are 12 for each method. Source data are provided as a Source Data file.

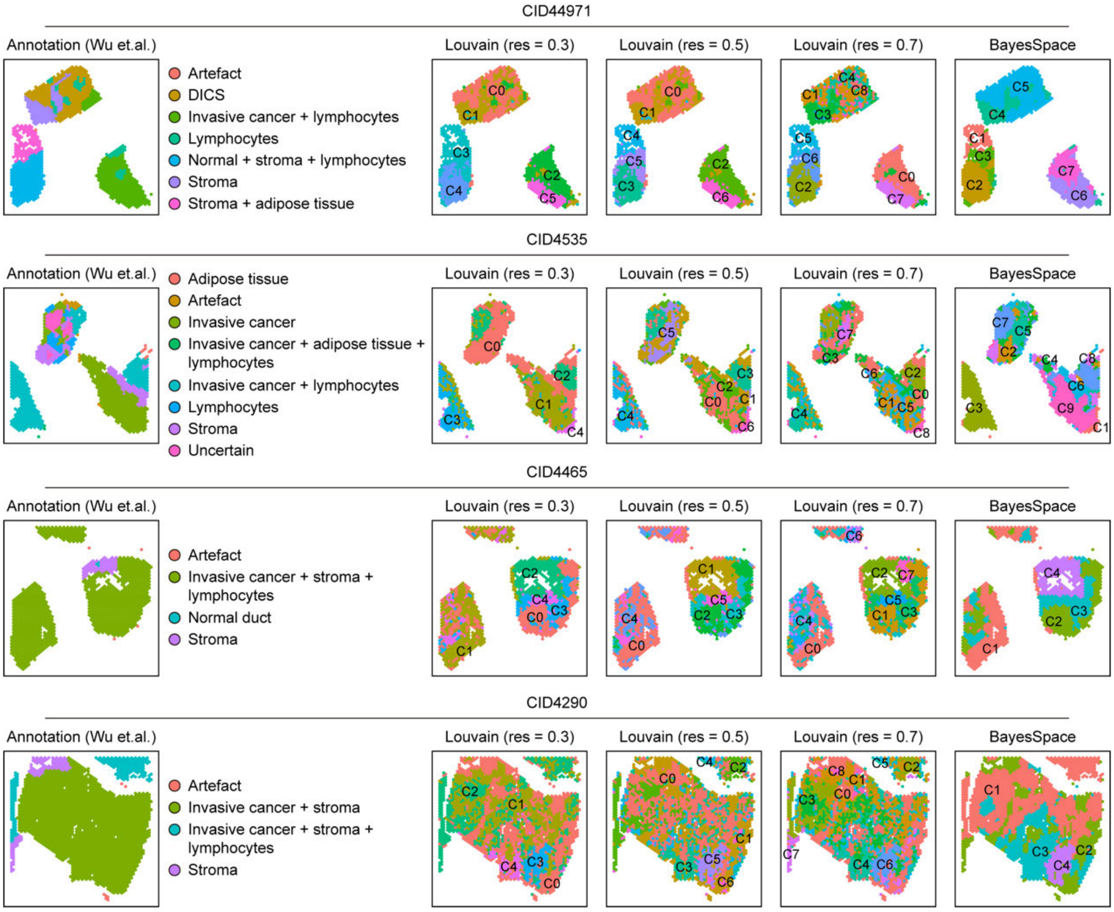

Supplementary Figure S36. The 10X Visium SRT data of breast cancer generated by Wu et.al. The spots were colored with the pathological annotation provided by the authors, the unsupervised clustering results with different resolutions, and the spatial domain clustering results.

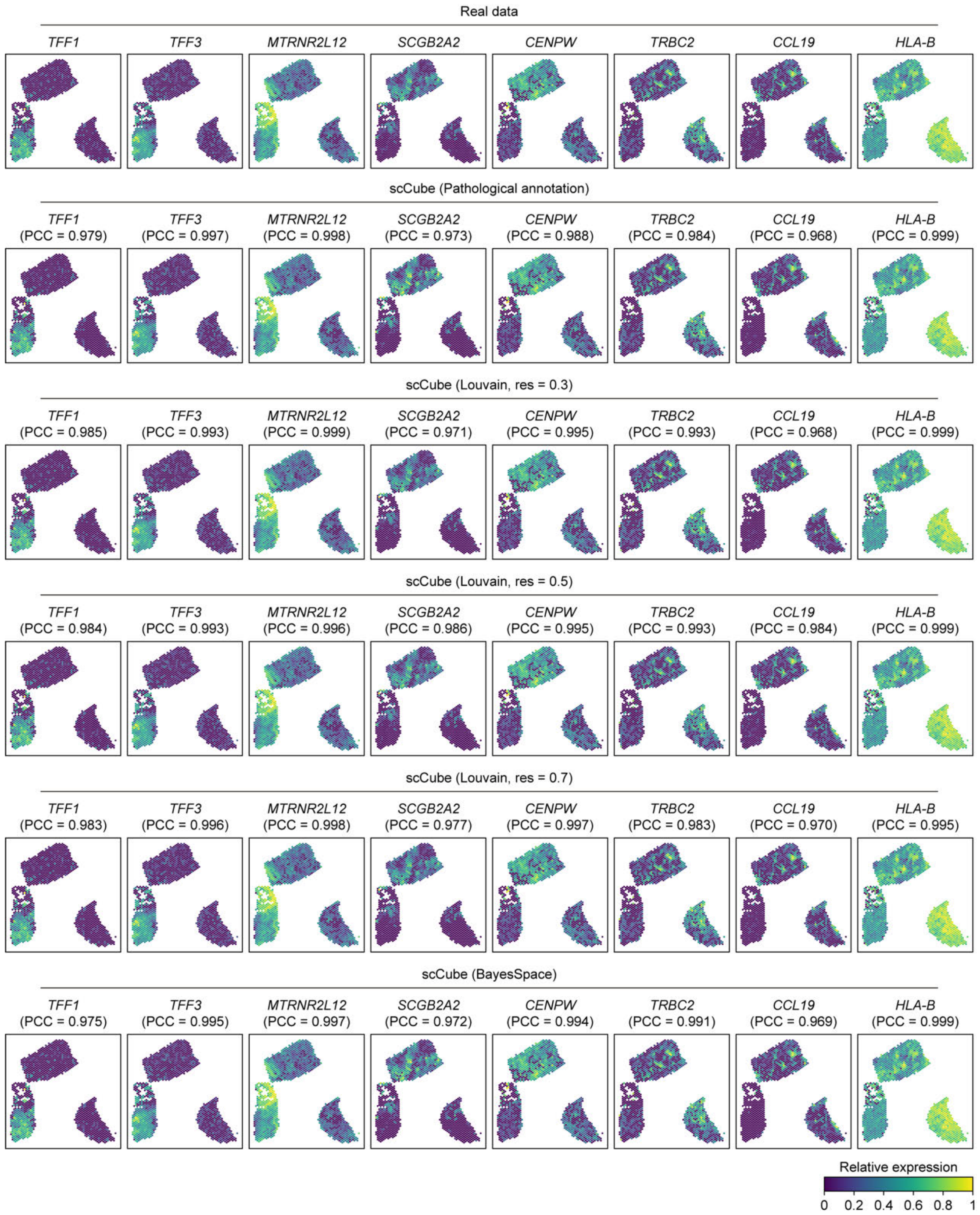

Supplementary Figure S37. The spatial expression patterns of eight representative genes in the real data (CID44971) and the simulated data generated by scCube using different annotation information. Source data are provided as a Source Data file.

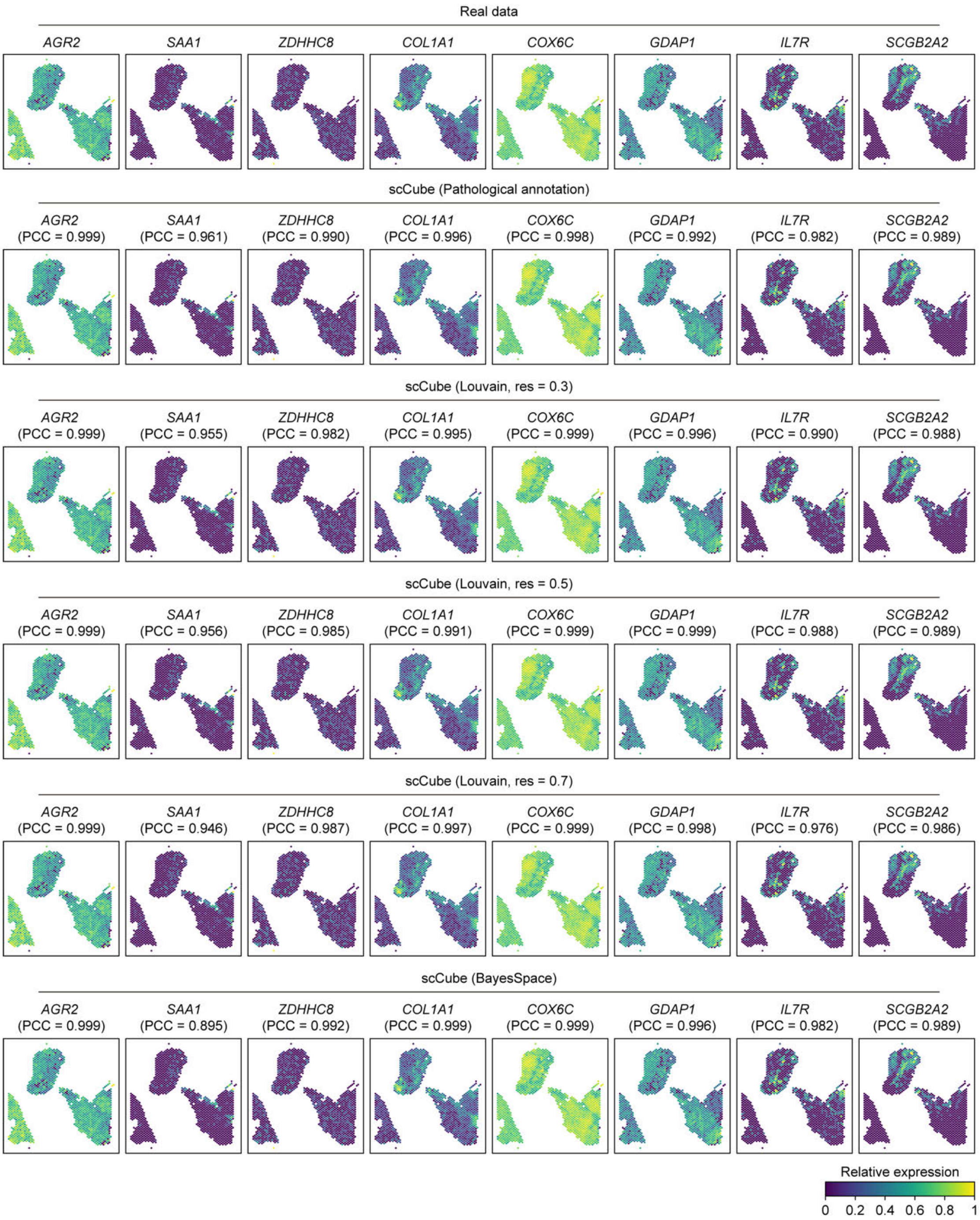

Supplementary Figure S38. The spatial expression patterns of eight representative genes in the real data (CID4535) and the simulated data generated by scCube using different annotation information. Source data are provided as a Source Data file.

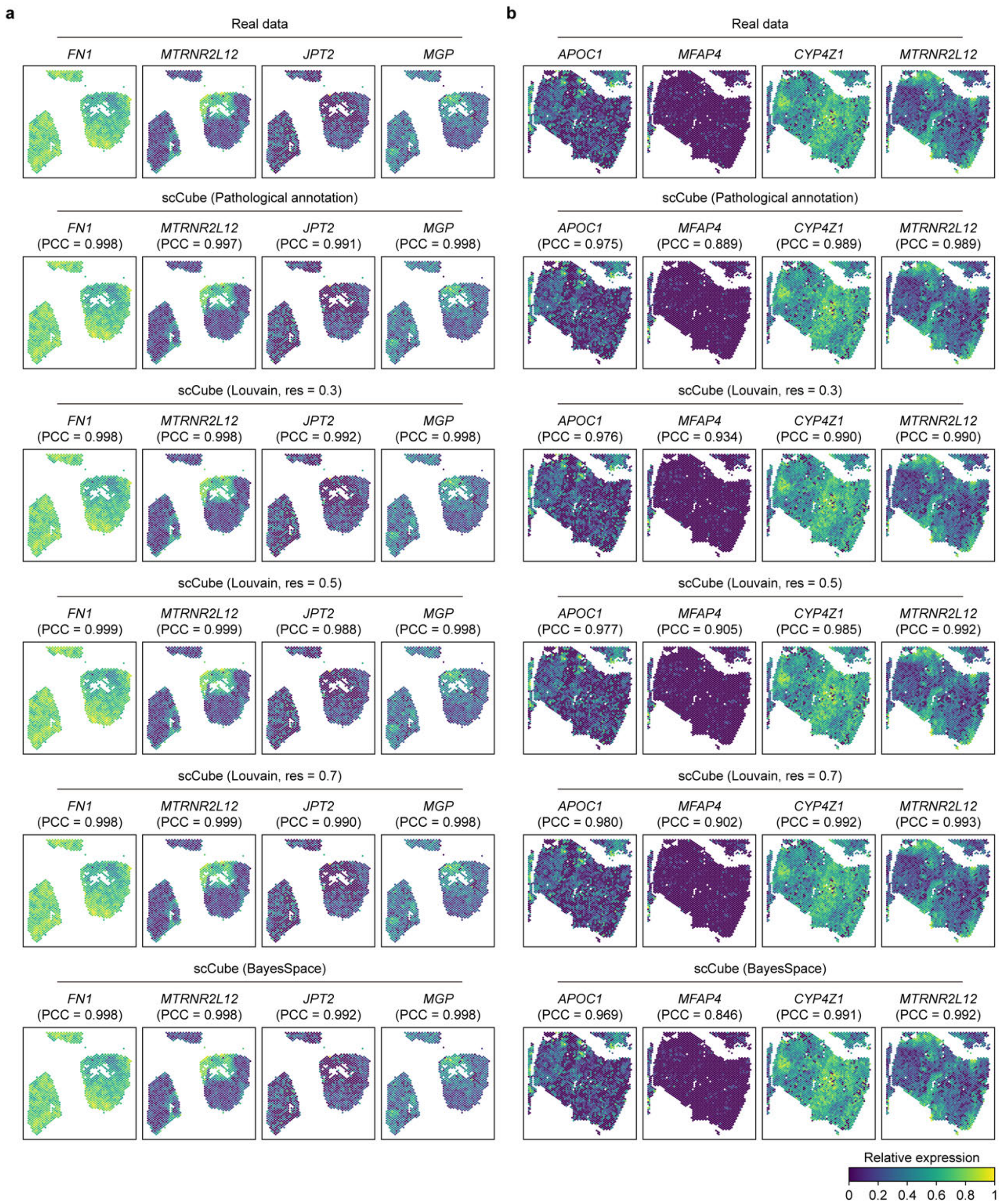

Supplementary Figure S39. The spatial expression patterns of four representative genes in the real data (a, CID4465; b, CID4290) and the simulated data generated by scCube using different annotation information. Source data are provided as a Source Data file.

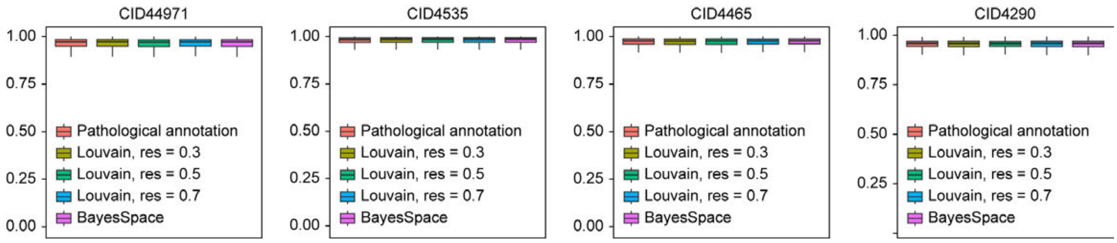

Supplementary Figure S40. Boxplots of PCC values between the real spatial expression patterns of all genes and the corresponding simulated spatial expression patterns across four breast cancer 10X Visium SRT datasets. The simulated data were generated by scCube using different annotation information. Data are presented as boxplots (minima, 25th percentile, median, 75th percentile, and maxima). The number of data points are 19,237 for each dataset. Source data are provided as a Source Data file.

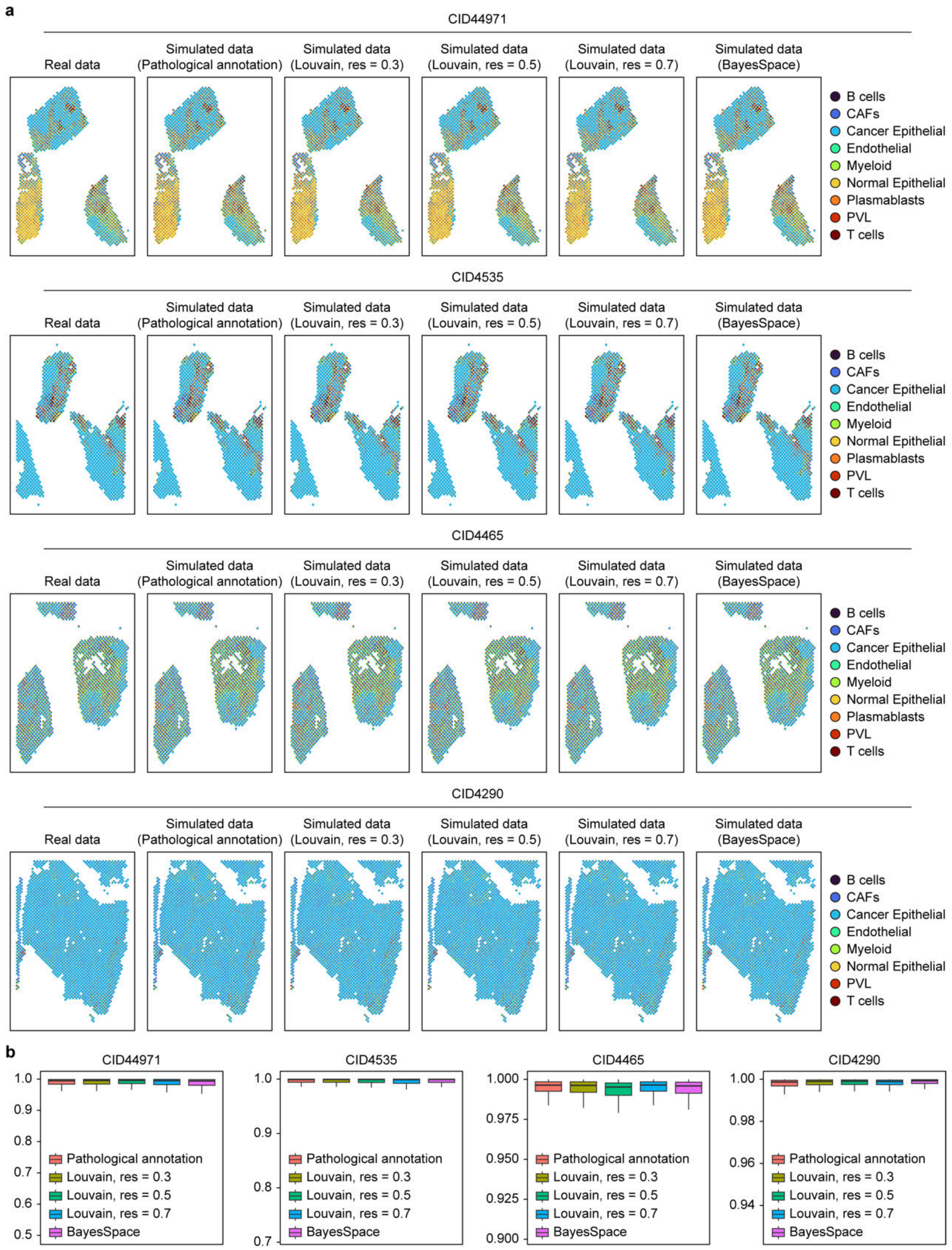

Supplementary Figure S41. Performance evaluation of scCube based on the spot deconvolution results. a, The deconvolution results by RCTD on the real and scCube-simulated data. **b,** Boxplots of PCC values between the deconvolution results on the real and scCube-simulated data. Data are presented as boxplots (minima, 25th percentile, median, 75th percentile, and maxima). The number of data points are 1,160, 1,124, 1,211, and 2,426 for CID44971, CID4535, CID4465, and CID4290, respectively. Source data are provided as a Source Data file.

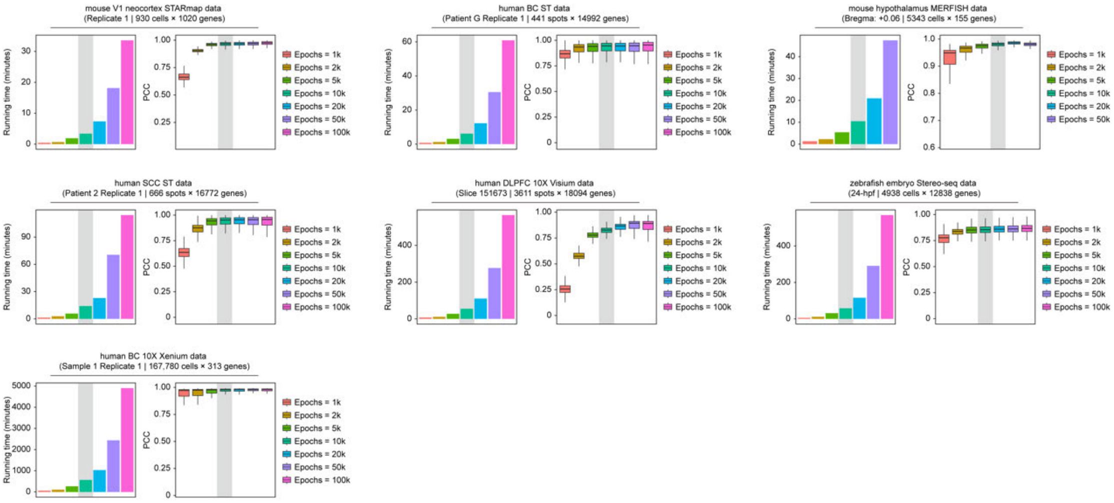

Supplementary Figure S42. The execution time and simulation performance of scCube with different number of training epochs across seven benchmark datasets of different sizes. The grey background highlights the number of training epochs (10,000 epochs) when scCube reach a relatively stable simulation performance. Data are presented as boxplots (minima, 25th percentile, median, 75th percentile, and maxima). The number of data points are 1,020, 14,992, 155, 16,772, 18,094, 12,838, and 313 for mouse V1 neocortex STARmap data, human BC ST data, mouse hypothalamus MERFISH data, human SCC ST data, human DLPFC 10X Visium data, zebrafish Stereo-seq data, and human BC 10X Xenium data, respectively. Source data are provided as a Source Data file.

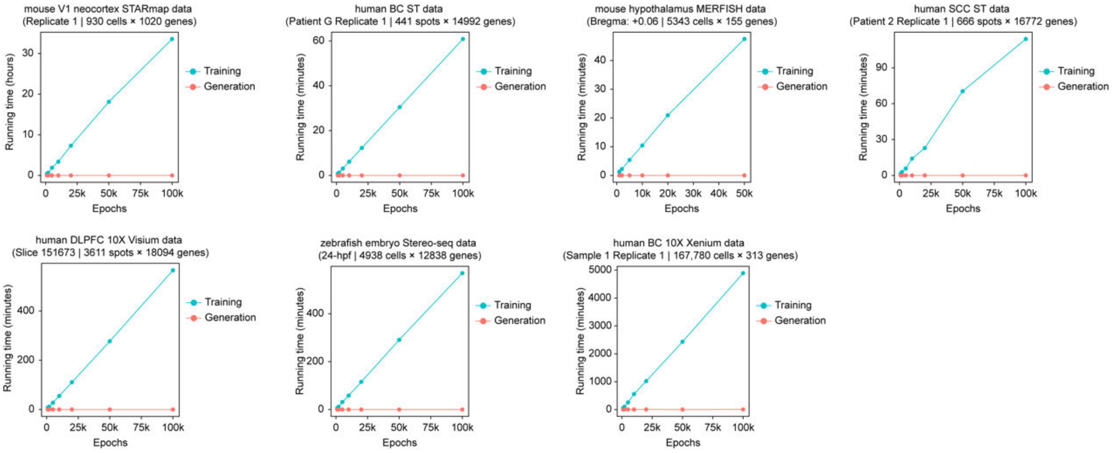

Supplementary Figure S43. The execution time of training and generation steps of scCube with different number of training epochs across seven benchmark datasets of different sizes. Source data are provided as a Source Data file.

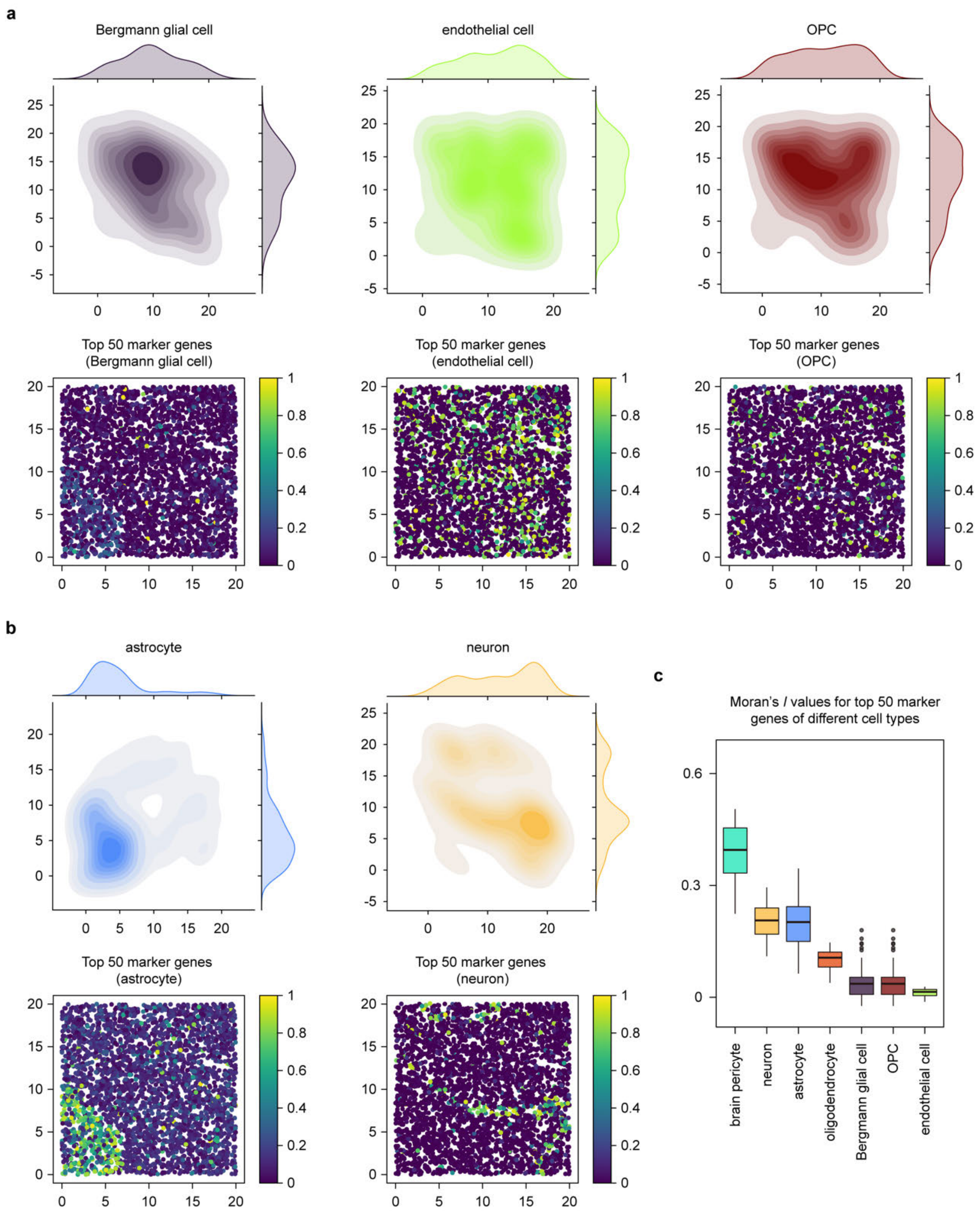

Supplementary Figure S44. scCube generates spatial patterns with diverse types and numbers. a, The spatial distributions of other three cell types that are set as "without spatial patterns" (top) and the spatial expression patterns for top 50 marker genes of each cell type (bottom). **b,** The spatial distributions of other two cell types that are set as "with spatial patterns" (top) and the spatial expression patterns for top 50 marker genes of each cell type (bottom).**c,** Boxplots of Moran's *I* values for the spatial expression patterns of top 50 marker genes of different cell types. Data are presented as boxplots (minima, 25th percentile, median, 75th percentile, and maxima). The number of data points are 50 for each cell type. Source data are provided as a Source Data file.

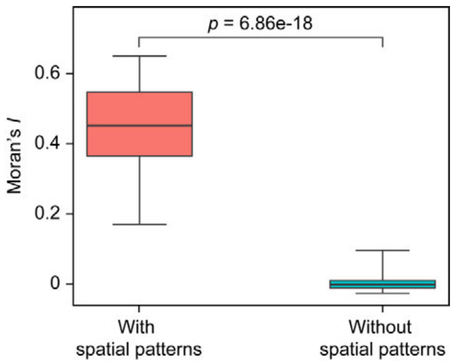

Supplementary Figure S45. Boxplots of Moran's *I* values for spatial expression patterns of marker genes of astrocytes in two simulated datasets. The P-value is calculated with the two-sided Wilcoxon rank-sum test. Data are presented as boxplots (minima, 25th percentile, median, 75th percentile, and maxima). The number of data points are 50 for genes with and without spatial patterns. Source data are provided as a Source Data file.

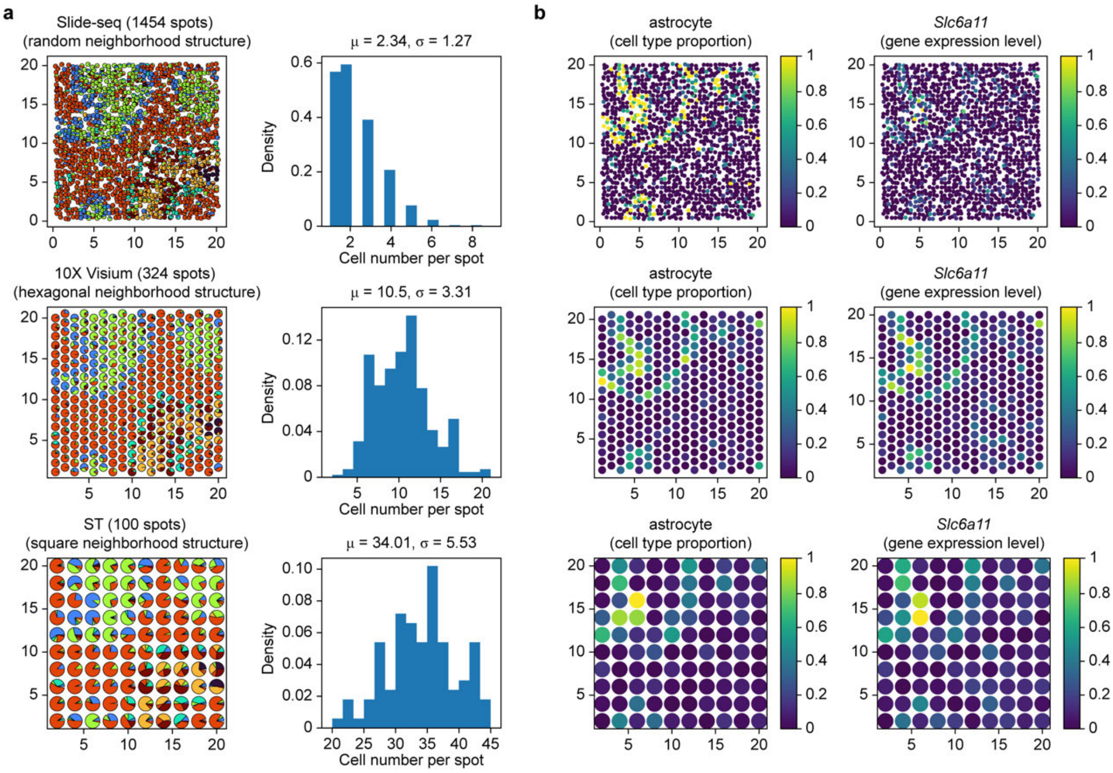

Supplementary Figure S46. scCube generates spot-based spatial patterns with diverse resolutions and spot arrangements. a, The simulated spatial patterns with diverse spot arrangements generated by scCube (left) and frequency histograms of the cell number per spot (right). **b,** The spatial distribution of astrocyte (left) and spatial expression pattern of *Slc6a11* (right) in three simulated spatial patterns with diverse spot arrangements. Source data are provided as a Source Data file.

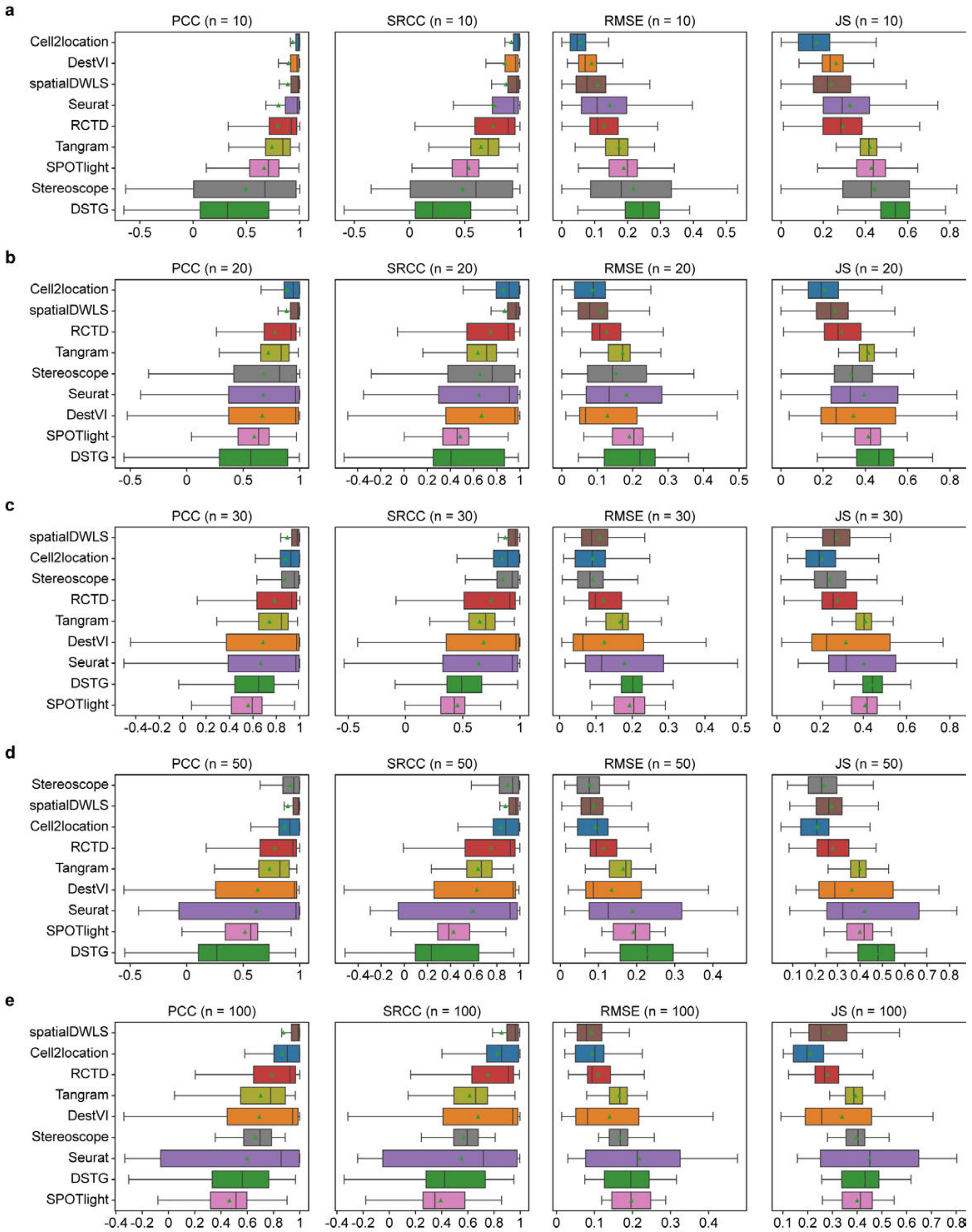

Supplementary Figure S47. Using scCube to benchmark spot deconvolution methods. Boxplots of PCC, SSIM, RMSE, and JS values of the nine spot deconvolution methods for resolution (n=10) (**a**), resolution (n=20) (**b**), resolution (n=30) (**c**), resolution (n=50) (**d**), and resolution (n=100) (**e**) simulated SRT datasets. Data are presented as boxplots (minima, 25th percentile, median, 75th percentile, and maxima). The number of data points of each method are 961, 484, 324, 196, and 100 for resolution (n=10) (**a**), resolution (n=20) (**b**), resolution (n=30) (**c**), resolution (n=50) (**d**), and resolution (n=100) (**e**) simulated SRT datasets, respectively. Source data are provided as a Source Data file.

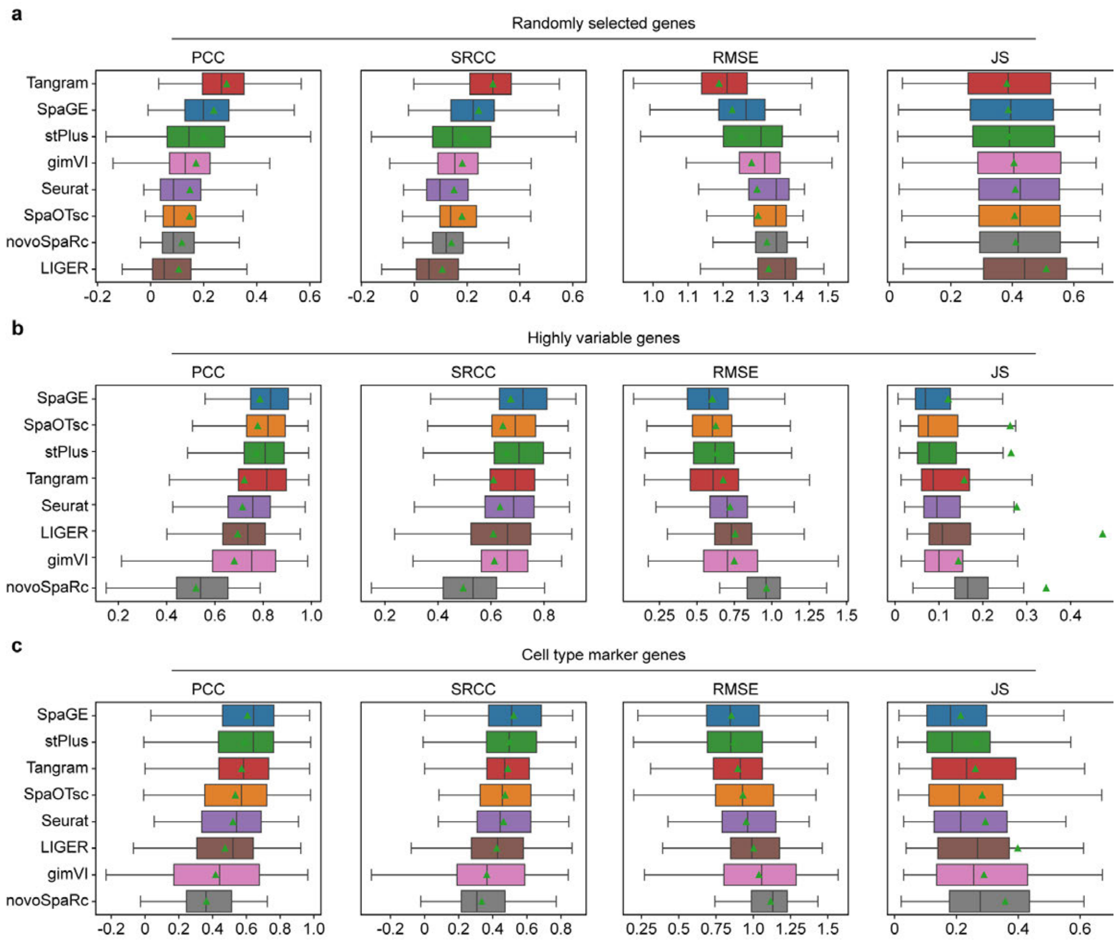

Supplementary Figure S48. Using scCube to benchmark gene imputation methods. Boxplots of PCC, SSIM, RMSE, and JS values of the eight gene imputation methods for simulated SRT datasets with randomly selected genes (**a**), highly variable genes (**b**), and cell type marker genes (**c**). Data are presented as boxplots (minima, 25th percentile, median, 75th percentile, and maxima). The number of data points are 200 for each type of targeted genes of each method. Source data are provided as a Source Data file.

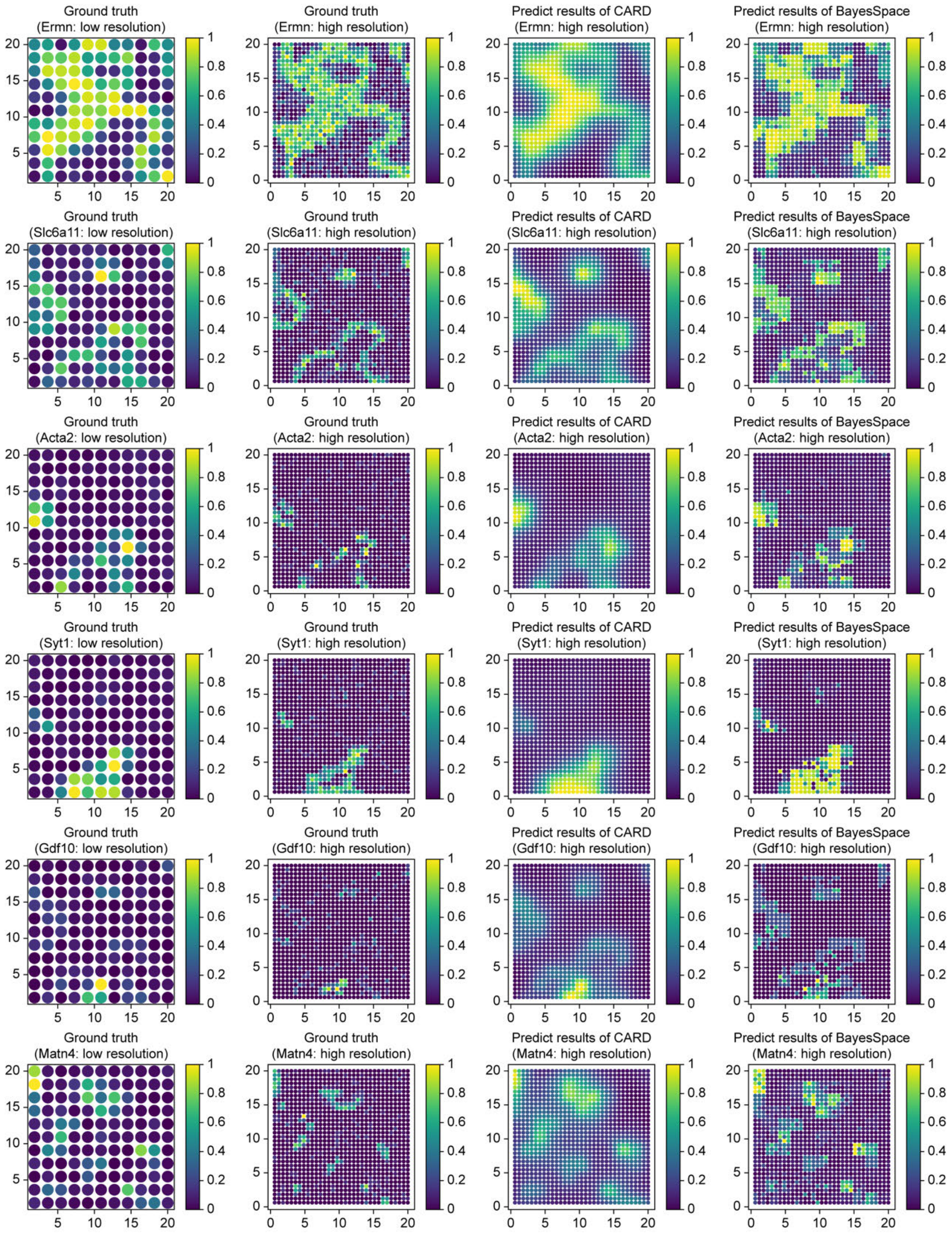

Supplementary Figure S49. Using scCube to benchmark resolution enhancement. The high-resolution spatial maps of *Ermn*, *Slc6a11*, *Acta2*, *Syt1*, *Gdf10*, and *Matn4* constructed by CARD and BayesSpace are showed respectively.

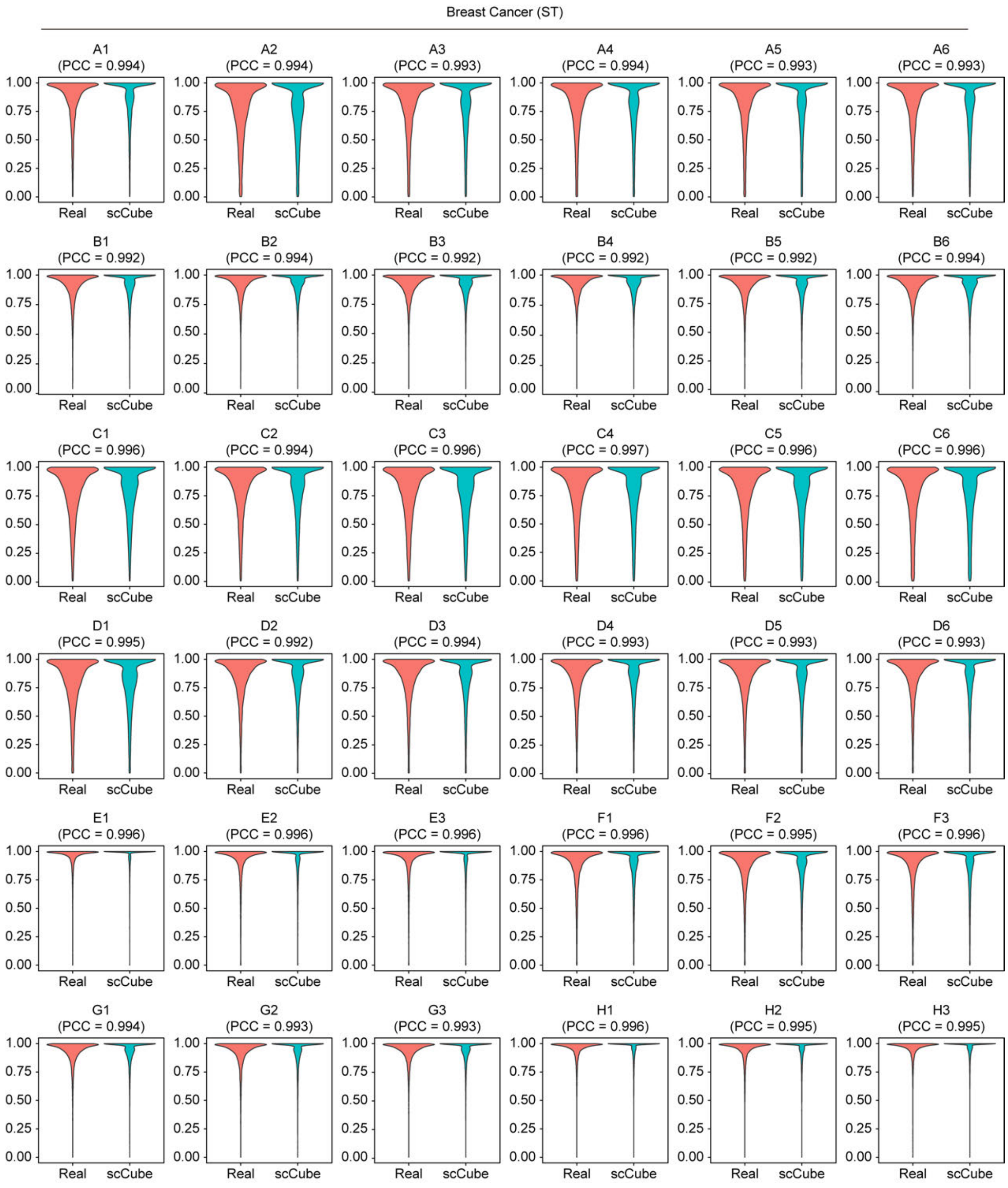

Supplementary Figure S50. Sparsity comparison between real data and scCube-simulated data across 36 human breast cancer ST data. Source data are provided as a Source Data file.

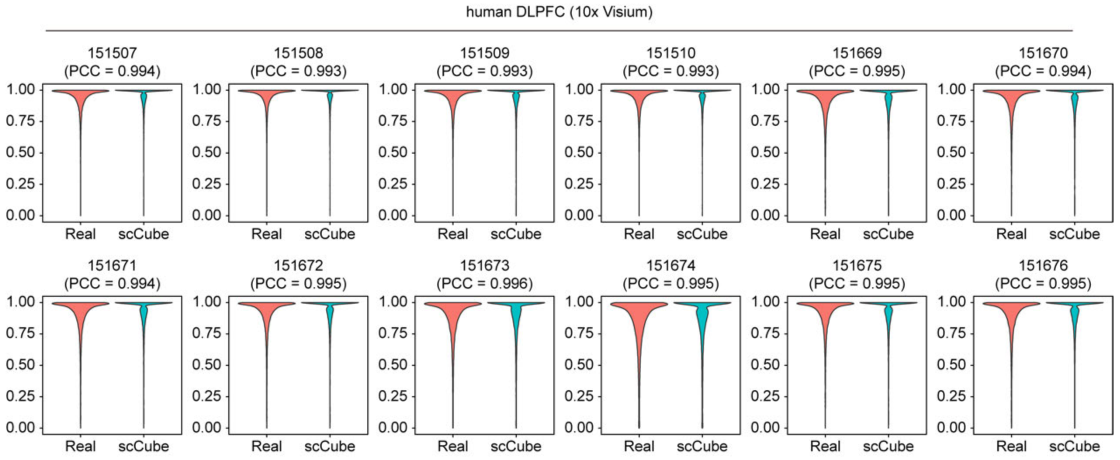

Supplementary Figure S51. Sparsity comparison between real data and scCube-simulated data across 12 human DLPFC 10X Visium data. Source data are provided as a Source Data file.

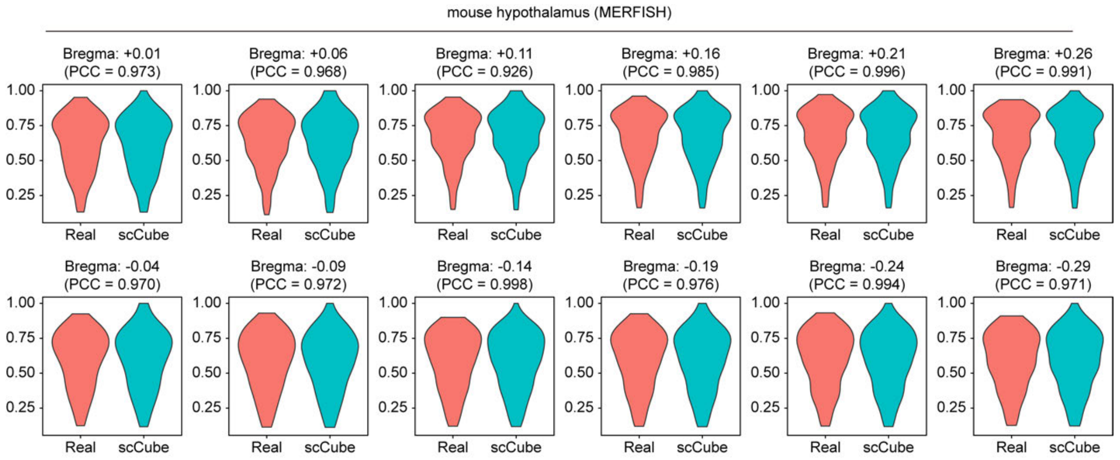

Supplementary Figure S52. Sparsity comparison between real data and scCube-simulated data across 12 mouse hypothalamus MERFISH data. Source data are provided as a Source Data file.

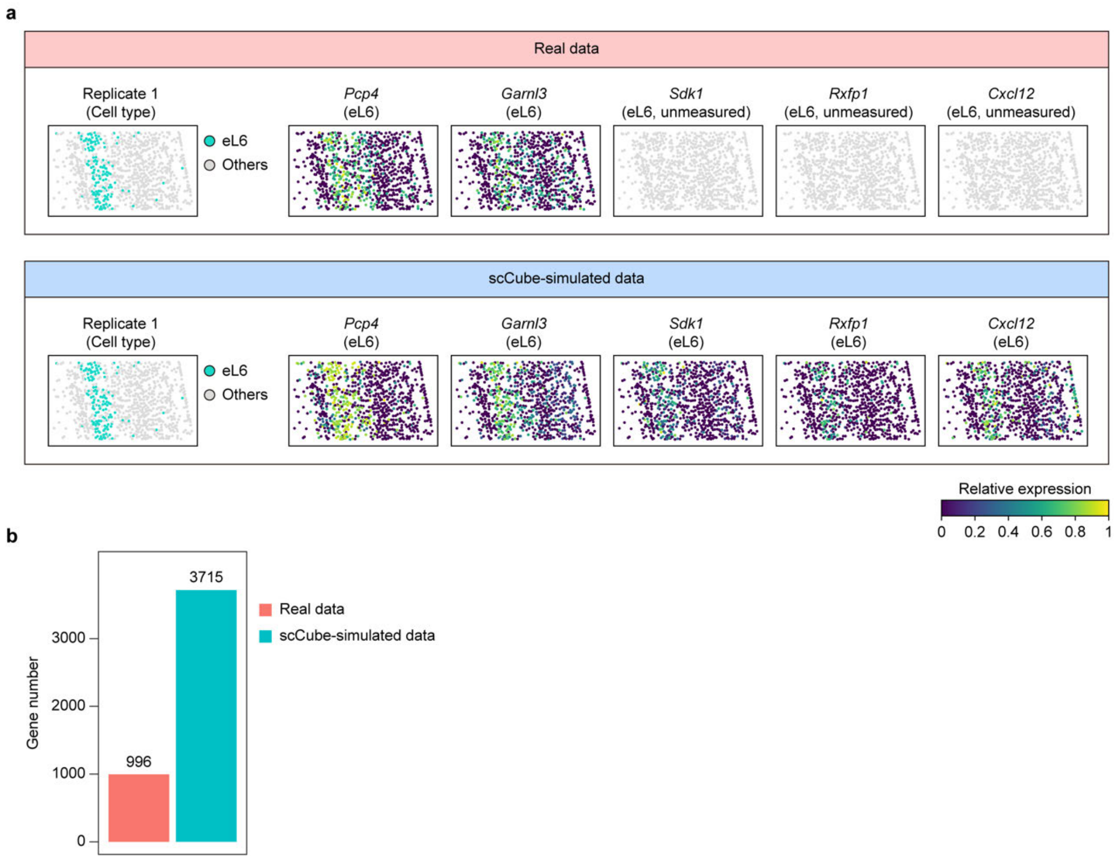

Supplementary Figure S53. scCube generates simulated SRT data with various gene numbers. a, The scCube-simulated data provides additional "ground truth" of the gene expression values for unmeasured genes compared with the real data. **b,** The number of genes in the real and scCube-simulated data.

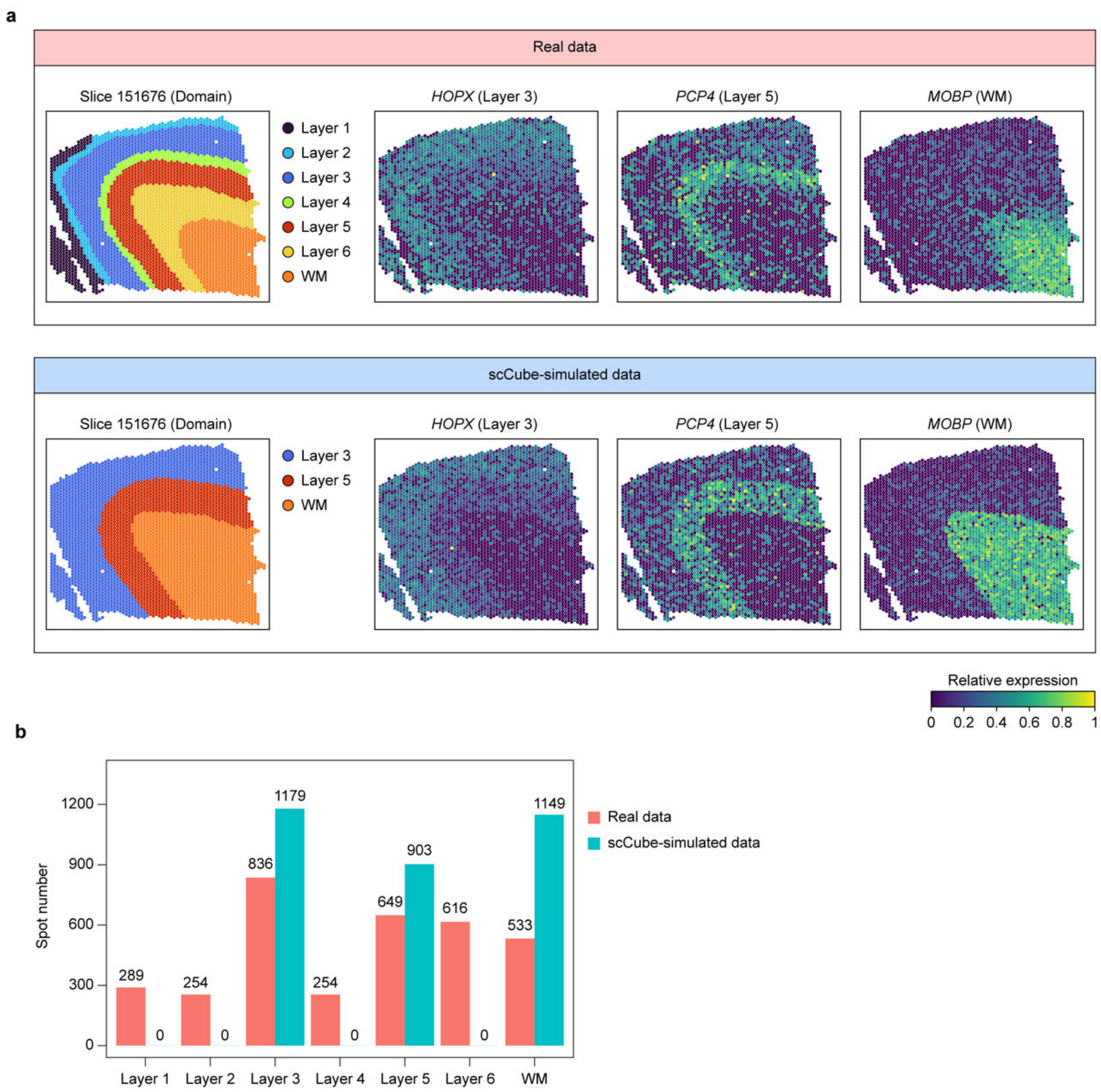

Supplementary Figure S54. scCube generates simulated SRT data with various spatial pattern numbers and sizes. a, The scCube-simulated data provides additional "ground truth" of the spatial patterns compared with the real data. **b,** The number of spots of each domain in the real and scCube-simulated data.

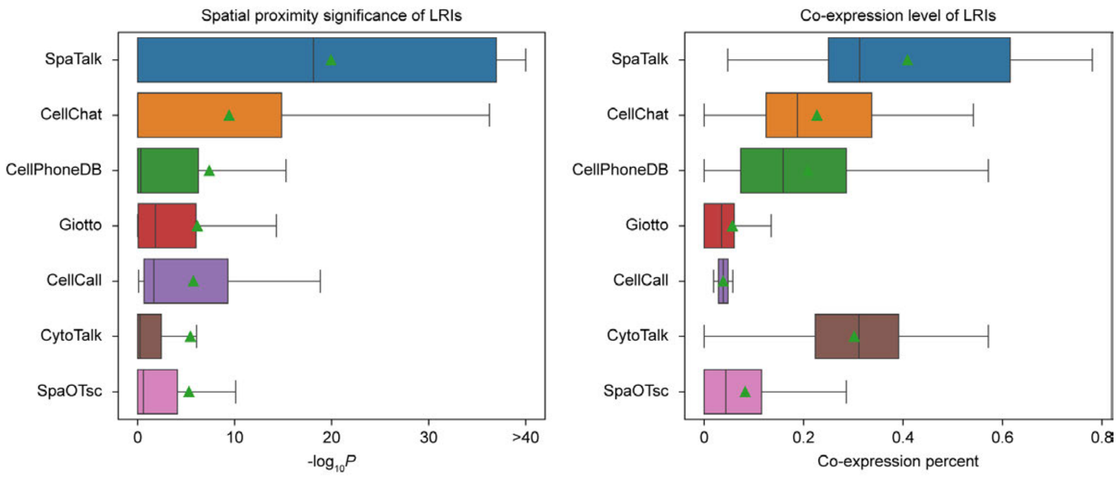

Supplementary Figure S55. Using scCube to benchmark spatial cell-cell interaction inference methods. To evaluate the accuracy of different methods in spatial cell-cell interaction inference, the spatial proximity significance and the co-expression level on the cell-cell spatial graph network of the ligand–receptor interactions (LRIs) inferred by each method are compared. Data are presented as boxplots (minima, 25th percentile, median, 75th percentile, and maxima). The number of data points (spatial proximity significance of LRIs) are 21, 210, 1000, 119, 6, 103, and 1000 for SpaTalk, CellChat, CellPhoneDB, Giotto, CellCall, CytoTalk, and SpaOTsc, respectively; the number of data points (co-expression level of LRIs) are 21, 27, 82, 16, 2, 7, and 81 for SpaTalk, CellChat, CellPhoneDB, Giotto, CellCall, CytoTalk, and SpaOTsc, respectively. Source data are provided as a Source Data file.

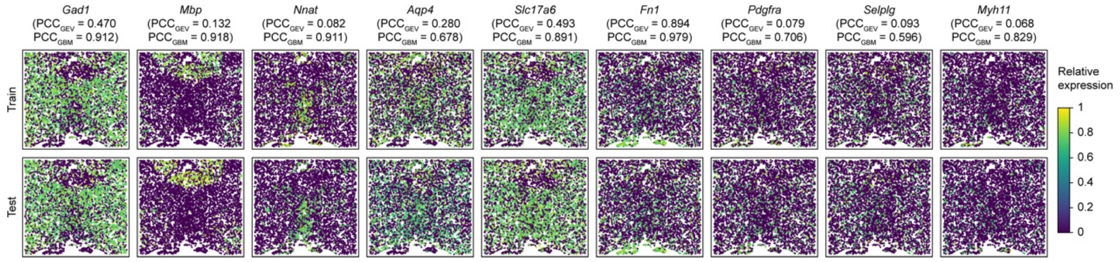

Supplementary Figure S56. Comparison of two evaluation metrics for spatial patterns. The training dataset and test dataset is split from the mouse hypothalamus MERFISH data (Bregma: +0.06) using countsplit. PCC_{GEV}: the Pearson correlation coefficient values between the gene expression vector for each gene across spatial positions in real and simulated data. PCC_{GBM}: the Pearson correlation coefficient values between the two GBMs' predicted gene expression values from the simulated data's spatial locations.
