# Supplementary material for: Simulating multiple variability in spatially resolved transcriptomics with scCube
Source: Nat Commun. 2024 Jun 12;15:5021. doi: 10.1038/s41467-024-49445-0 (PMC11169532; doi:10.1038/s41467-024-49445-0)
Supplement: Supplementary file 3 — Description of Additional Supplementary Files [file 41467_2024_49445_MOESM3_ESM.pdf]

Description of Additional Supplementary Files:

Supplementary Data 1: Detailed information of SRT datasets used in trained models of scCube.

Supplementary Data 2: Detailed information of SRT datasets used in performance comparison.

Supplementary Data 3: Detailed information of SRT datasets used in overfitting issues evaluation.
