# Supplementary material for: Simulating multiple variability in spatially resolved transcriptomics with scCube
Source: Nat Commun. 2024 Jun 12;15:5021. doi: 10.1038/s41467-024-49445-0 (PMC11169532; doi:10.1038/s41467-024-49445-0)
Supplement: Supplementary file 7 — Reporting Summary [file 41467_2024_49445_MOESM7_ESM.pdf]

nature portfolio

Corresponding author(s): Xiaohui Fan, Yiyu Cheng

Last updated by author(s): Mar 20, 2024

Reporting Summary

Nature Portfolio wishes to improve the reproducibility of the work that we publish. This form provides structure for consistency and transparency in reporting. For further information on Nature Portfolio policies, see our Editorial Policies and the Editorial Policy Checklist.

Statistics

For all statistical analyses, confirm that the following items are present in the figure legend, table legend, main text, or Methods section.

n/a	Confirmed	
☐	☒	The exact sample size (*n*) for each experimental group/condition, given as a discrete number and unit of measurement
☐	☒	A statement on whether measurements were taken from distinct samples or whether the same sample was measured repeatedly
☐	☒	The statistical test(s) used AND whether they are one- or two-sided<br>*Only common tests should be described solely by name; describe more complex techniques in the Methods section.*
☒	☐	A description of all covariates tested
☐	☒	A description of any assumptions or corrections, such as tests of normality and adjustment for multiple comparisons
☐	☒	A full description of the statistical parameters including central tendency (e.g. means) or other basic estimates (e.g. regression coefficient) AND variation (e.g. standard deviation) or associated estimates of uncertainty (e.g. confidence intervals)
☒	☐	For null hypothesis testing, the test statistic (e.g. *F*, *t*, *r*) with confidence intervals, effect sizes, degrees of freedom and *P* value noted<br>*Give P values as exact values whenever suitable.*
☒	☐	For Bayesian analysis, information on the choice of priors and Markov chain Monte Carlo settings
☒	☐	For hierarchical and complex designs, identification of the appropriate level for tests and full reporting of outcomes
☐	☒	Estimates of effect sizes (e.g. Cohen's *d*, Pearson's *r*), indicating how they were calculated

Our web collection on statistics for biologists contains articles on many of the points above.

Software and code

Policy information about availability of computer code

Data collection: No software was used for data collection

Data analysis: The scCube package is available at https://github.com/ZJUFanLab/scCube/.

Specific package version used for analysis are as follows (python 3.8.5):
anndata==0.8.0
numpy==1.23.5
pandas==1.5.3
scanpy==1.9.1
pot==0.8.2
matplotlib==3.6.3
seaborn==0.12.2
tqdm==4.64.1
torch==1.12.0
cell2location==0.1.3
DSTG==0.0.1
Stereoscope==03
Tangram==1.0.4
SpaGE

stPlus==0.0.6
scvi-tools==1.0.3
SpaOTsc==0.2
novoSpaRc==0.4.3
SpaGCN==1.2.7
STAGATE==1.0.0
stLearn==0.4.11
cellphonedb==2.1.7

Specific package version used for analysis are as follows (R 4.1.0):
SRTsim==0.99.6
scDesign3==0.99.1
scDesign2==0.1.0
spacexr==2.0.3
Seurat==4.2.0
Giotto==3.3.0
SymSim==0.0.0.9000
Splatter==1.20.0
SPOTlight==1.0.0
liger==1.0.0
BayesSpace==1.6.0
CARD==1.1
DR.SC==3.3
SpaTalk==1.0
CellCall==1.0.7
CellChat==1.6.1
CytoTalk==0.99.9
Giotto==1.1.2
nichenetr==2.0.4
countsplit==4.0.0
caret==6.0-94

For manuscripts utilizing custom algorithms or software that are central to the research but not yet described in published literature, software must be made available to editors and reviewers. We strongly encourage code deposition in a community repository (e.g. GitHub). See the Nature Portfolio guidelines for submitting code & software for further information.

Data

Policy information about availability of data

All manuscripts must include a data availability statement. This statement should provide the following information, where applicable:

- Accession codes, unique identifiers, or web links for publicly available datasets
- A description of any restrictions on data availability
- For clinical datasets or third party data, please ensure that the statement adheres to our policy

No experimental data conducted by ourselves are used. All data used in this study is publicly available and can be accessed through the following links:

(1) the human dorsolateral prefrontal cortex (DLPFC) 10X Visium dataset [http://spatial.libd.org/spatialLIBD/];
(2) the mouse hypothalamus MERFISH dataset [https://doi.org/10.5061/dryad.8t8s248];
(3) the mouse neocortex V1 STARmap dataset [https://zenodo.org/record/7830764#.ZDpObi-1HUI];
(4) the human HER2 breast cancer "Spatial Transcriptomics (ST)" dataset [https://zenodo.org/record/5511763#.Y6kMduxBzUI];
(5) the human skin squamous cell carcinoma (SCC) "Spatial Transcriptomics (ST)" dataset [GSE144240 (https://www.ncbi.nlm.nih.gov/geo/query/acc.cgi?acc=GSE144240)];
(6) the human breast cancer 10X Xenium dataset [https://www.10xgenomics.com/products/xenium-in-situ/preview-dataset-human-breast];
(7) the zebrafish embryo Stereo-seq dataset [https://gene.ai.tencent.com/SpatialOmics/dataset?datasetID=83];
(8) the human breast cancer 10X Visium and paired scRNA-seq dataset [https://doi.org/10.5281/zenodo.4739739];
(9) the he Tabula Muris scRNA-seq dataset [https://figshare.com/projects/Tabula_Muris_Transcriptomic_characterization_of_20_organs_and_tissues_from_Mus_musculus_at_single_cell_resolution/27733];
(10) the Tabula Sapiens scRNA-seq dataset [https://figshare.com/projects/Tabula_Sapiens/100973];
(11) the MCA scRNA-seq dataset [https://figshare.com/articles/MCA_DGE_Data/5435866];
(12) the HCL scRNA-seq dataset [https://figshare.com/articles/HCL_DGE_Data/7235471];
(13) the human TNBC Multiplexed Imaging (MIBI) channel images [https://mibi-share.ionpath.com]

Research involving human participants, their data, or biological material

Policy information about studies with human participants or human data. See also policy information about sex, gender (identity/presentation), and sexual orientation and race, ethnicity and racism.

Reporting on sex and gender	Reporting on sex and gender is not relevant to our study.
Reporting on race, ethnicity, or other socially relevant groupings	Reporting on race, ethnicity, or other socially relevant groupings are not relevant to our study.
Population characteristics	Population characteristics is not relevant to our study.
Recruitment	Recruitment is not relevant to our study.
Ethics oversight	Ethics oversight is not relevant to our study.

Note that full information on the approval of the study protocol must also be provided in the manuscript.

Field-specific reporting

Please select the one below that is the best fit for your research. If you are not sure, read the appropriate sections before making your selection.

- [x] Life sciences
- [ ] Behavioural & social sciences
- [ ] Ecological, evolutionary & environmental sciences

For a reference copy of the document with all sections, see nature.com/documents/nr-reporting-summary-flat.pdf

Life sciences study design

All studies must disclose on these points even when the disclosure is negative.

Sample size	No new data was generated for this study. All data used in this study is publicly available, so no statistical method was used to predetermine sample size. The rationale for these sample sizes are based on previously published related papers, and we included more datasets to comprehensively benchmark our method than the original studies. We analyzed 29 publicly available SRT datasets from seven different tissues generated by six sequencing technologies that include 10X Visium, ST, Stereo-seq, MERFISH, STARmap, and 10X Xenium. We also analyzed 4 publicly available scRNA-seq datasets, including Tabula Muris, Tabula Sapiens, Mouse Cell Atlas (MCA), and Human Cell Landscape (HCL). All these results show that scCube is able to maintain robustness with different types of data.
Data exclusions	There were no data exclusions except for the filtering of 5 “blank” barcodes and the Fos gene in the MERFISH data.
Replication	We benchmarked scCube on 29 publicly available SRT datasets from seven different tissues generated by six sequencing technologies that include 10X Visium, ST, Stereo-seq, MERFISH, STARmap, and 10X Xenium. Our conclusions are consistent from these replications, which demonstrated that scCube is robust in simulating the SRT data.
Randomization	Randomization is not relevant to our study. The primary objective of our research is to simulate the SRT data, which does not involve statistical modeling with covariates. Moreover, our method does not involve assigning treatments. As a result, randomization is unnecessary for our study.
Blinding	Blinding is not relevant to our study because no new data collection was involved in the present study.

Reporting for specific materials, systems and methods

We require information from authors about some types of materials, experimental systems and methods used in many studies. Here, indicate whether each material, system or method listed is relevant to your study. If you are not sure if a list item applies to your research, read the appropriate section before selecting a response.

Materials & experimental systems

n/a	Involved in the study	
x		Antibodies
x		Eukaryotic cell lines
x		Palaeontology and archaeology
x		Animals and other organisms
x		Clinical data
x		Dual use research of concern
x		Plants

Methods

n/a	Involved in the study	
x		ChIP-seq
x		Flow cytometry
x		MRI-based neuroimaging
